# Revisiting potential associations between brain morphology, fear acquisition and extinction through new data and a literature review

**DOI:** 10.1038/s41598-020-76683-1

**Published:** 2020-11-16

**Authors:** Mana R. Ehlers, Janne Nold, Manuel Kuhn, Maren Klingelhöfer-Jens, Tina B. Lonsdorf

**Affiliations:** 1 Department of Systems Neuroscience, University Medical Center Hamburg-Eppendorf, Martinistrasse 52, W34, 20246 Hamburg, Germany; 2 Department of Psychiatry, Harvard Medical School, and Center for Depression, Anxiety and Stress Research, McLean Hospital, Belmont, MA 02478 USA

**Keywords:** Neuroscience, Psychology

## Abstract

Inter-individual differences in defensive responding are widely established but their morphological correlates in humans have not been investigated exhaustively. Previous studies reported associations with cortical thickness of the dorsal anterior cingulate cortex, insula and medial orbitofrontal cortex as well as amygdala volume in fear conditioning studies. However, these associations are partly inconsistent and often derived from small samples. The current study aimed to replicate previously reported associations between physiological and subjective measures of fear acquisition and extinction and brain morphology. Structural magnetic resonance imaging was performed on 107 healthy adults who completed a differential cued fear conditioning paradigm with 24 h delayed extinction while skin conductance response (SCR) and fear ratings were recorded. Cortical thickness and subcortical volume were obtained using the software Freesurfer. Results obtained by traditional null hypothesis significance testing and Bayesians statistics do not support structural brain-behavior relationships: Neither differential SCR nor fear ratings during fear acquisition or extinction training could be predicted by cortical thickness or subcortical volume in regions previously reported. In summary, the current pre-registered study does not corroborate associations between brain morphology and inter-individual differences in defensive responding but differences in experimental design and analyses approaches compared to previous work should be acknowledged.

## Introduction

Marked inter-individual differences in defensive responding have been suggested to be the result of underlying neurobiological differences that manifest as stable trait-like characteristics (rodents^1^, humans^2^). Defensive conditioned responding can be investigated in the laboratory by means of fear conditioning protocols.

Generally, the fear conditioning procedure comprises different experimental phases^2^: throughout acquisition training an innately aversive stimulus, the unconditioned stimulus (US), is paired with an initially neutral stimulus, the conditioned stimulus (CS+), producing a conditioned response (CR) to the CS+ while a second control stimulus (CS−) is never paired with the US. Hence, a fear memory is formed as the CS+ gains predictive power of the appearance of a US and comes to elicit a defensive conditioned response by itself. In the laboratory, different outcome measures such as skin conductance response (SCR), fear potentiated startle (FPS), ratings of fear and/or US expectancy as well as BOLD fMRI can be used as proxies thereof. The difference in responding to the CS+ and the CS− (i.e., CS discrimination) is taken as an approximation for the strength of fear learning. During extinction training, the CS+ is no longer coupled with the US and a plethora of results suggest that an inhibitory extinction memory is formed as a consequence^3^. As a result, conditioned responding is reduced. When at a later time exposed to the CS+ (i.e., ‘retention test’ or ‘return of fear test’, for an overview see^3^) one can either observe a ‘retention of the extinction memory’ indicating dominance of the extinction memory or the return of conditioned responding (i.e., ‘return of fear’) indicating dominance of the fear over the extinction memory.

While the basic mechanisms of fear conditioning and extinction and the importance of inter-individual differences in defensive responding are well recognized, research concerning a potential mapping of such inter-individual differences onto variability in brain morphology is sparse. Structural-brain-behavior associations (i.e., associations between inter-individual variability in brain morphology and behavior or physiology) have a long history in psychology and neuroscience^4,5^. In in vivo human studies inter-individual variability in brain structure is commonly extracted from anatomical scans acquired through magnetic resonance imaging (MRI). The most common methods include measures of grey matter tissue such as grey matter volume using the Computational Anatomy Toolbox^6^ and measures of cortical thickness and subcortical volume using the software Freesurfer^7–10^. Yet, structural-brain-behavior associations were recently scrutinized as it was shown in a large sample of healthy adults that significant associations are rare and also show low replication rates across a range of psychological measures^11–13^.

Previous work in fear conditioning research has reported individual differences in brain morphology to be associated with differences in conditioned responding during fear and extinction learning as well as retention of extinction. Most of these studies have focused on skin conductance response (SCR) while fewer studies investigated associations with ratings of valence, arousal or CS–US contingency awareness^14–16^ and a single study with fear potentiated startle^17^ (see Table 1). Of note, all areas that have been reported to show structural associations with inter-individual differences in defensive responding during fear and/or extinction learning have been linked to group averages in functional brain activation as assessed by BOLD fMRI during learning and expression of fear and extinction^18,19^: the amygdala, insula and prefrontal areas (dorsal anterior cingulate cortex (dACC), and medial orbitofrontal cortex (mOFC) in cue conditioning as well as the hippocampus in context conditioning.

**Table 1:** Experimental design overview of studies investigating associations between brain morphology and associative processes during fear acquisition training and extinction in human participants.

References	N	Segmenta-tion approach	RIR (%)	Extinction	# of Acq trials for CS+/CS−	# of Ext trials for CS+/CS−	Outcome measures		Tested associations with				SCR quanti-fication via	SCR scoring criteria; CS duration	Covariates
							SCR	Fear rating	CSdiff	CS+	CS−	CSavg			
Abend et al., 2019^17^	250	Freesurfer	80	Immediate	10/10	8/8	✓	✓	✗	✗	✗	✓	TTP	0–5 s post CS onset; 7 s CS	Age, anxiety
Abend et al., 2020^22^	351	Freesurfer	80	N/A	10/10	8/8	✓	✓	✗	✓^a^	✗	✗	TTP	1–5 s post CS onset; 7 s CS	Age, anxiety
Cacciaglia et al., 2013^14^	52	Manual	50	Immediate	36/36	18/18	✓ ✓	✓	✓	✓	✓	✗	TTP	1–9 s post CS onset; 6 s CS	Age, gender, anxiety, education
Ehlers et al. (current study)	107	Freesurfer	100	Delayed	14/14	14/14	✓	✓	✓	✓	✓	✗	TTP	0.9–3.5 s post CS onset; 6 s CS	TIV, sex
Hartley et al., 2011^20^	18	Freesurfer	17	N/A	21/15;	N/A	✓	✗	✓	✗	✗	✗	TTP	0.5–4.5 s post CS onset; 4 s CS	Sex, anxiety
	12	Freesurfer	35	Immediate	23/15	15/15	✓	✗	✓	✗	✗	✗	TTP	0.5–4.5 s post CS onset; 4 s CS	Sex, anxiety
Milad et al., 2005^24^	14	Freesurfer	100	Immediate	5/5	10/10	✓	✗	✓	✓	✓	✗	b.c.	Max (12 s post CS onset)-mean (2 s pre CS onset); 12 s CS	N/A
Milad et al., 2007^21^	14	Freesurfer	100	Immediate	5/5	10/10	✓	✗	✓	✗	✗	✗	b.c.	Max (12 s post CS onset)-mean (2 s pre CS onset); 12 s CS	N/A
Rauch et al., 2005^25^	14	Freesurfer	100	Immediate	5/5	10/10	✓	✗	✗	✓	✗	✗	b.c.	Max (12 s post CS onset)-mean (2 s pre CS onset); 12 s CS	Sex, extraversion, neuroticism
Winkelmann et al., 2015^16^	68; 53	Freesurfer	50	Immediate	36/36	18/18	✓	✓	✓	✗	✗	✗	Ledalab	Sum (SCRs 1–7 s post CS onset); 6 s CS	TIV, age, gender

Two studies (Abend et al. 2019, Abend et al. 2020) that did not investigate associative processes during fear acquisition training but average responding to the CS+ and CS− across experimental phases are included for completeness.

None of the studies explicitly instructed the participants with regard to the CS/US contingencies, Abend et al. (2019) and Hartley et al. (2011), however, informed participants about the fact that association can be learning during the experiment.

*RIR *reinforcement rate, *N/A *information not available, *CSdiff *differential SCR [(CS+) – (CS−)], *CSavg *SCR averaged across the CS+ and CS− as well as across fear acquisition and extinction training, *TTP *trough to peak, *b.c. *baseline correction, *TIV *total intracranial volume.

^a^In Abend et al. (2020) computational modeling of SCR to the CS+ was used to predict SCR over the course of learning and assess learning rate during acquisition and extinction.

Morphological variability within the amygdala has been positively related to average differential responding during acquisition training in SCR [(CS+) – (CS−)] but not ratings of arousal and valence^16^ or CS–US contingency^14,16^. More precisely, this association was reported for the volume of the *right* amygdala during early acquisition in sample 1 but during late acquisition in sample 2 despite the identical experimental protocol^16^ while a smaller earlier study with a largely overlapping sample reported a positive correlation with *left* amygdala volume in early but not late acquisition^14^. These discrepancies might be explained by differences in analyses such as segmentation approaches, different SCR quantification approaches, different scoring criteria for SCR as well as the inclusion of a large number of covariates as well as correction of differential SCR responding by responding during preceding experimental phases (see Table 1). In contrast to these studies, others did not find significant positive associations between differential [(CS+) − (CS−)] autonomic conditioned responding during acquisition training and amygdala volume but report an insignificant *negative* relationship in two small samples for both right and left amygdala^20^. It should be noted however that due to different numbers of trials included in these studies (see Table 1), the full acquisition phase in this study, corresponds largely to the first half of acquisition training in the studies by Cacciaglia et al.^14^ and Winkelmann et al.^16^.

In addition to the amygdala, the volume in the right posterior insula/posterior operculum was reported to show a positive association with differential SCR during fear acquisition training in two samples—although this did not survive correction for multiple comparisons in the smaller sample^20^.

Furthermore, SCR to the CS+, but not to the CS− or differential SCR responding during acquisition training were reported to be *positively* correlated with thickness of the dorsal anterior cingulate cortex (dACC)^21^ in a 100% reinforcement protocol which was, however, not replicated in two samples in a study employing partial reinforcement^20^. A recent study^17^ with a large sample of anxiety patients and healthy controls (N = 351) including children and adults, reported the dorsomedial/dorsolateral prefrontal cortex (dm/dlPFC)—a region located substantially more lateral than the area identified by Milad et al.^21^—to be *negatively* correlated with a measure of general SCR averaged across both CS types (i.e., CS+ and CS−) and experimental phases (i.e., fear acquisition and extinction training). The interpretation of this aggregate SCR measure, however, is not straightforward with respect to associative learning processes. While Abend et al. interpret the observed association in terms of aberrant threat and safety learning, alternatively this aggregate measure may as well reflect general arousal or the reactivity in SCR independent of associative learning processes. Future studies employing experimental paradigms to capture generalization of fear may clarify whether the association reported by Abend et al. could also be interpreted in terms of fear generalization. In another publication, computational modeling was applied to SCR to the CS+ which reveal that learning rate correlates positively with cortical thickness of the ventromedial prefrontal cortex (vmPFC), dACC and anterior insula^22^. In addition to these findings from cue-conditioning studies, a positive association between total hippocampal but not amygdala volume and differential second interval but not first interval SCR, see^23^ was reported during context conditioning^15^. No significant associations were observed with SCR during extinction^15^. In this study, CS–US contingency awareness showed a relationship with total brain volume, but not hippocampal or amygdala volume. Another study from the same research group reported an association between bilateral hippocampus volume and differential CS–US contingency ratings in late but not early acquisition training in a cue-conditioning paradigm—which seemed to be driven by a negative correlation with ratings to the CS−^14^. Differences in results might be attributable to the fact that the studies used a contextual and a cued fear conditioning paradigm respectively.

While the work summarized above focused on acquisition training, some studies have also investigated structural brain-behavior associations during extinction training and extinction retention. During immediate, early but not late extinction training, differential [(CS+) – (CS−)] SCR was correlated with the thickness of three clusters of the right vmPFC^16^. Notably, however, earlier studies^20,24^ did not test for any associations between prefrontal thickness and differential SCR during extinction training as they focused on 24 h extinction retention.

During a 24-delayed retention test (also often referred to as ‘extinction recall’, for a discussion on terminology see^4^), a positive correlation between a non-differential (i.e., CS+ specific) ‘extinction retention index’ in SCR and thickness of the medial OFC was observed when tested in the extinction context (i.e. ‘extinction retention’^23^) as well as in the mOFC portion of the vmPFC when tested in both the acquisition (i.e., ‘renewal’) and extinction context (i.e. ‘extinction retention’^22^). Another study reported a positive correlation between conditioned responding during a retention test and thickness of the vmPFC at a very lenient statistical threshold of *p* < 0.003 uncorrected following extinction training but not following cognitive regulation^20^. These studies^20,24,25^ quantified (extinction) retention in SCR through versions of the non-differential (i.e., CS+ specific) “Extinction retention index (ERI)”, which has recently been challenged from both a theoretical and empirical perspective for lacking construct validity: More specifically, non-differential ERIs likely measure general arousal or orienting responding rather than associative processes such as the retention of extinction memory^26^. As these studies did not investigate brain morphological associations during the preceding extinction training phase, the specificity of the findings pertaining to the retention test phase also remains unclear—in particular given the reported associations between volume in ventromedial prefrontal areas and differential SCR responding during extinction training itself—which always precedes a retention test.

To date, only a limited number of studies has linked inter-individual differences in brain morphology in areas known to be generally implicated in fear acquisition, extinction and extinction recall to conditioned autonomic (SCR) and subjective (valence and arousal ratings, fear ratings and CS–US awareness) measures of defensive responding.

**Figure 1:**
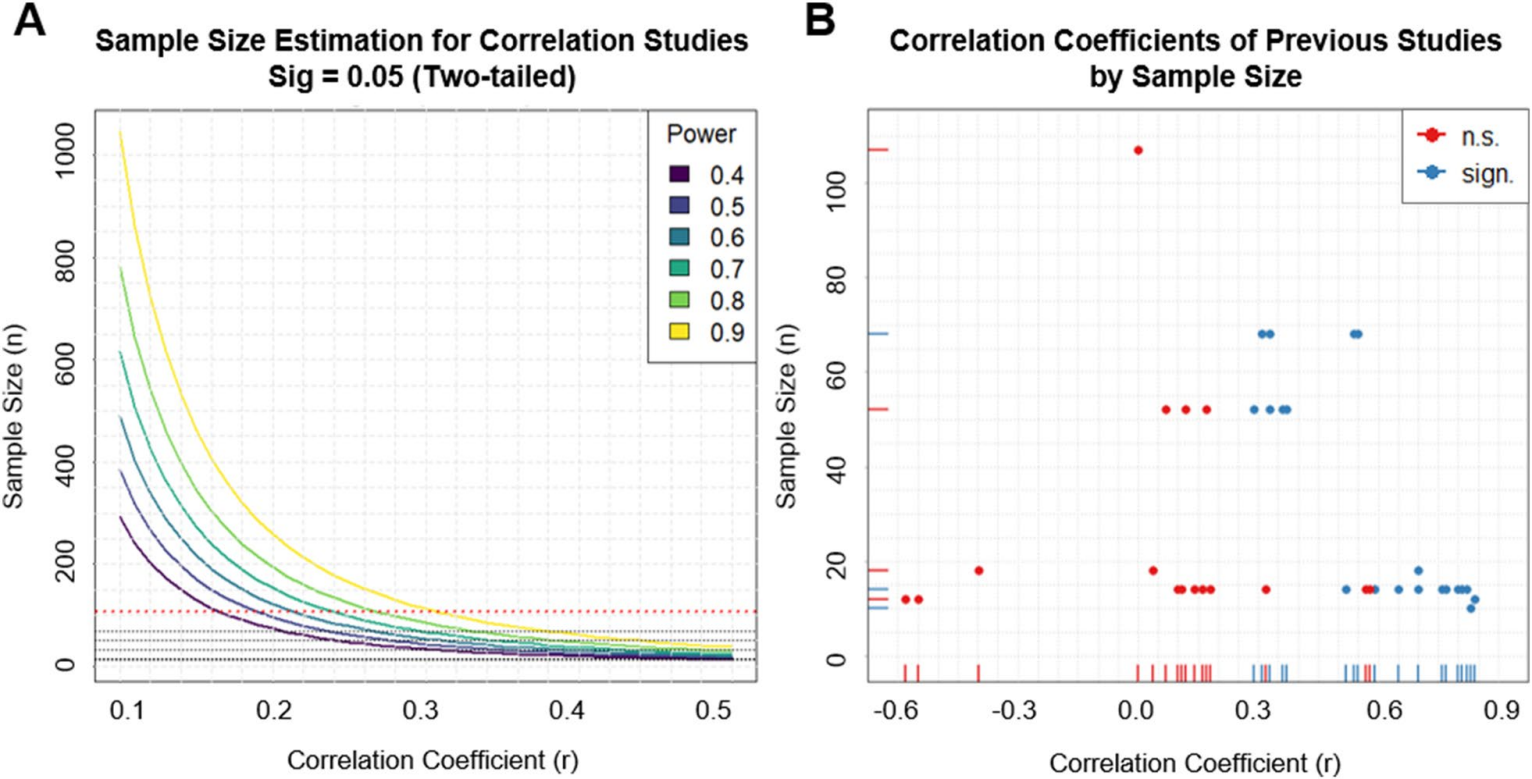
Illustration of (**A**) estimated power of correlation studies given a certain sample size. Dotted grey lines indicate sample sizes of previous studies investigating differential or CS-specific associations with brain morphological measures. Note that in some previous studies regressions were performed rather than correlations. For comparability all effects size measures were transformed to correlation coefficients. The red line indicates the sample size of the current study. (**B**) Effect sizes expressed as correlation coefficients obtained in previous studies and the current study plotted by sample size. Note that in some previous studies regressions were performed instead of correlation. For comparability all effects size measures were transformed to correlation coefficients. Red dots indicate non-significant and blue dots indicate significant findings. Note that some studies report more than one association and are hence represented with multiple dots.

From the perspective of the research standards in 2020, particularly the early studies report rather implausibly high correlation coefficients (illustrated in Fig. 1A) and partly employ (very) lenient statistical thresholds originating from what now has to be considered massively underpowered sample sizes^27,28^ (see Fig. 1B for a power curve plot) with only 10–18 participants^20,21,24,25^ (see Table 1).

While structural MRI measures themselves have been shown to have excellent reliability^28,29^ (minimal test–retest reliability 0.82^30^), the robustness of structural brain-behavior associations in general has been challenged recently^5,13^ and given this, the aim of the current pre-registered study (10.17605/osf.io/y73qw) is to replicate previously reported associations between individual differences in brain morphology and physiological (i.e., SCR) and subjective (i.e., fear ratings) measures of defensive responding during fear acquisition and delayed extinction in a larger sample of healthy adults (N = 107). More precisely, we aim to investigate previously reported associations between the cortical thickness of the dACC and insula as well as amygdala volume during acquisition training and the association between amygdala volume and mOFC thickness and extinction.

## Results

### Main effects of task

**Figure 2:**
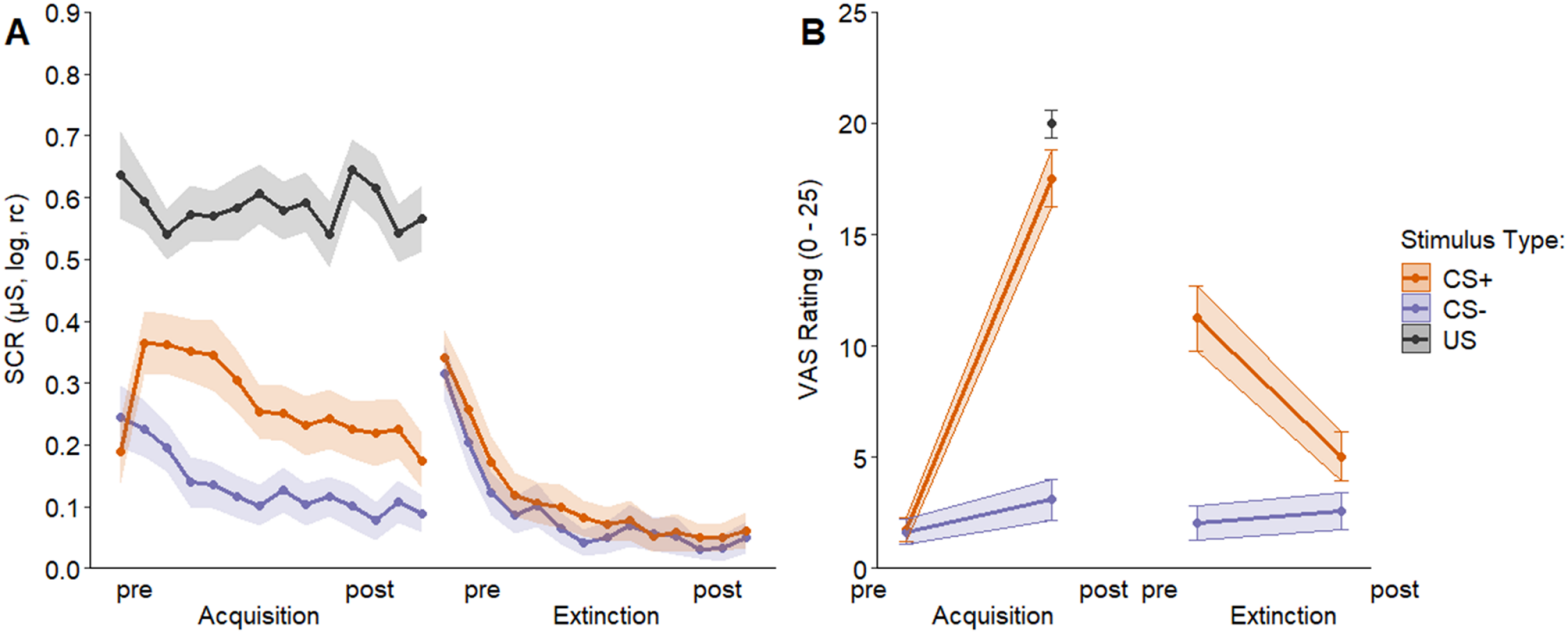
(**A**) SCR to the CS+ as compared to the CS− as well as the US during acquisition and extinction training (illustrated trial-by-trial) as well as (**B**) fear ratings in response to CS+ and CS− prior to and after fear acquisition training and extinction as well as aversiveness ratings to the US after acquisition training. 95% confidence intervals are illustrated by coloured bands. Note that a linear learning process is not assumed, the lines are meant to facilitate the visualization of the general trend from pre to post acquisition and extinction.

Successful acquisition training is reflected in significantly larger average SCR (see Fig. 2A) elicited by the CS+ than those elicited by the CS− (*t*(106) = 12.81, *p* < 0.001, 95% CI [0.11, 0.15]). Similarly, ratings of fear, anxiety and tension (see Fig. 2B) were significantly higher to the CS+ relative to the CS− after acquisition training (*t*(102) = 19.74, *p* < 0.001, 95% CI [13.08, 16.00]).

During extinction, the CS+, on average, still elicited larger SCR (see Fig. 2A) prior to extinction as compared to the CS− (*t*(106) = 3.94, *p* < 0.001, 95% CI [0.01, 0.03]). At the end of extinction, however (last five trials in SCR for both CS types), SCR elicited by the CS+ and CS− did not differ significantly (*t*(106) = 1.57, *p* = 0.12, 95% CI [− 0.003, 0.024]).

For extinction, a two-way ANOVA for fear ratings revealed a main effect of time (pre vs post extinction) (*F*(1, 412) = 25.06, *p* < 0.001), a main effect of CS type (*F*(1, 412) = 108.65, *p* < 0.001) as well as a significant interaction (*F*(1, 412) = 37.45, *p* < 0.001). Pairwise comparisons showed that the CS+ elicited higher ratings relative to the CS− prior to extinction (ps < 0.001) as well post extinction (ps = 0.010). Extinction success indicated by fear ratings is however supported by the observation that ratings of the CS+ dropped significantly from pre to post extinction (ps < 0.001), but not for the CS− (ps = 0.892).

### Inter-hemispheric differences in cortical thickness and volume

**Figure 3:**
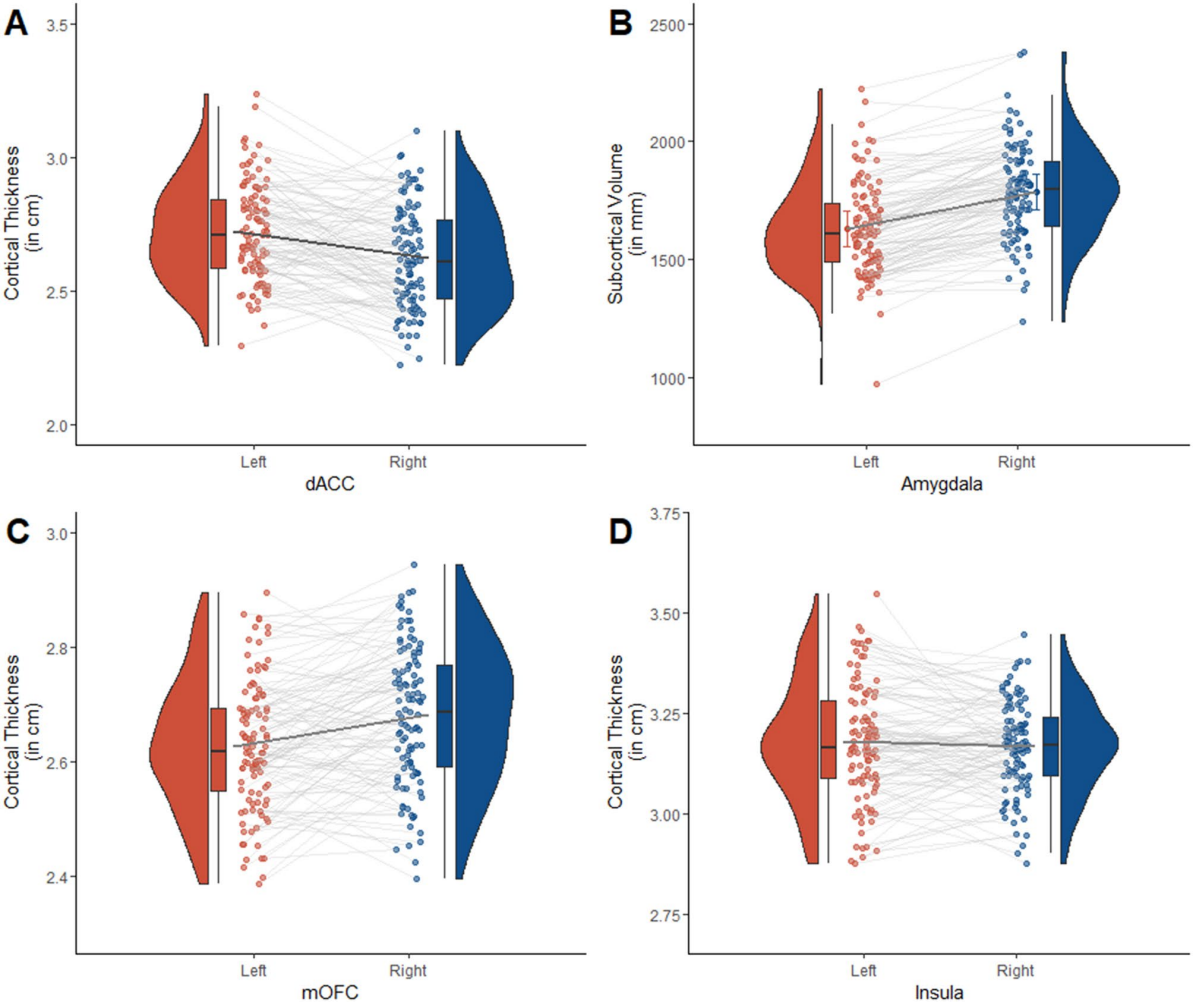
Illustration of (**A**) cortical thickness of the dACC, (**B**) volume of the amygdala, (**C**) cortical thickness of mOFC, and (**D**) cortical thickness of the insula in left (red) and right (blue) hemisphere. Data are illustrated by smoothed density distributions, individual subject means (dots), medians (boxplots) and interquartile ranges (boxes depict interquartile range and whiskers depict 1.5 × the interquartile range) for each hemisphere with data points derived from a single individual connected through grey lines.

Significant differences between volumina and cortical thickness in left and right hemisphere were observed for most regions: dACC (*t*(106) = 4.80, *p* < 0.001, *d* = 0.46), mOFC (*t*(106) = − 5.05, *p* < 0.001, *d* = 0.49) and amygdala (*t*(106) = − 14.89 , *p* < 0.001, *d* = 1.44) except for the insula (*t*(106) = 0.97, *p* = 0.33, *d* = 0.10) (see Fig. 3). Robustness analyses performing the main analyses reported here (see below) separately for the left and right hemisphere yielded comparable results (see Supplementary Material Section 2.1 for details).

### No association between brain morphology and indices of fear learning during acquisition and extinction training

**Figure 4:**
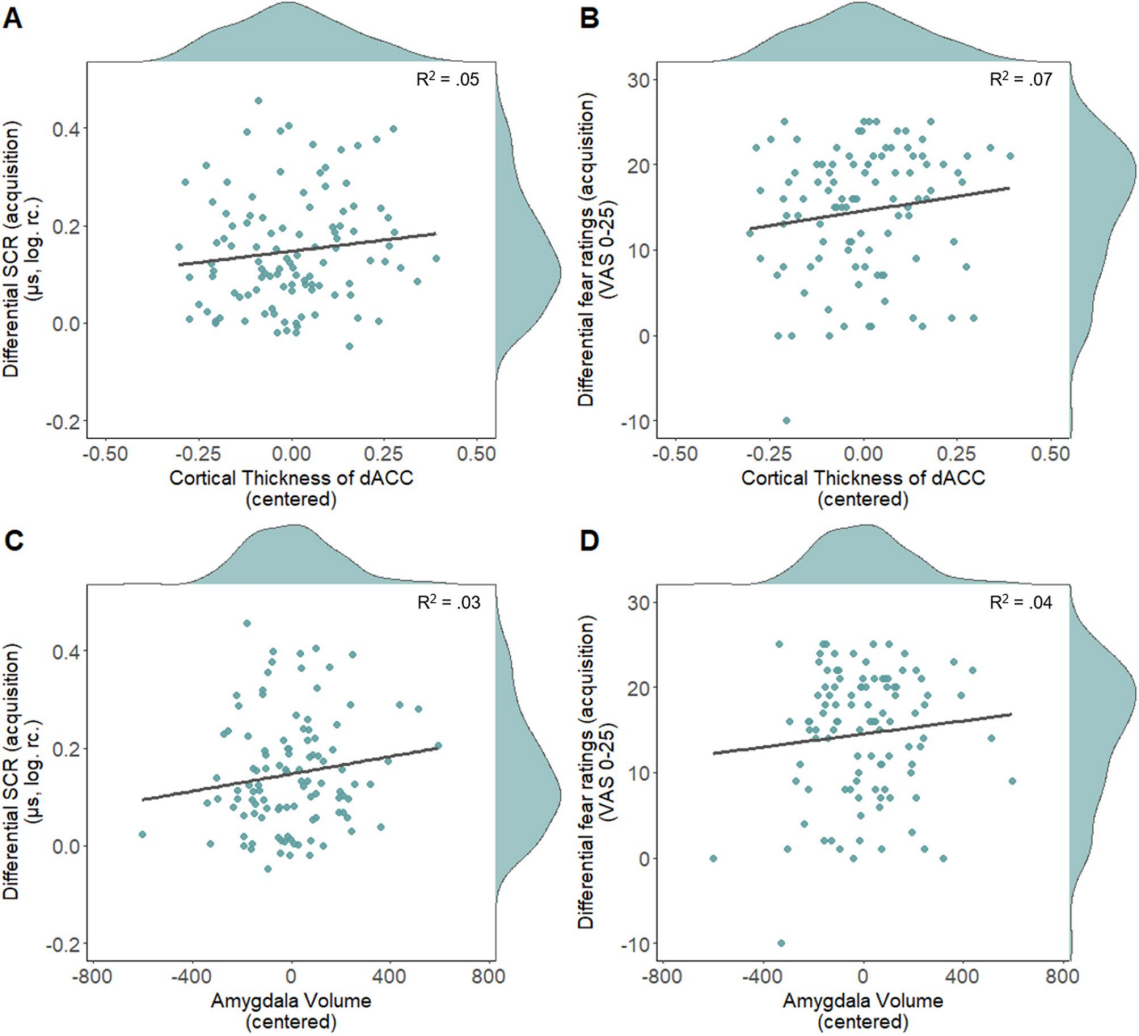
Scatterplots with marginal densities illustrating the (absence of) associations between average differential SCR [(CS+) − (CS−)] during acquisition training and (**A**) cortical thickness of the dACC and (**B**) the amygdala as well as between differential post acquisition fear ratings and (**C**) the dACC and (**D**) the amygdala.

**Figure 5:**
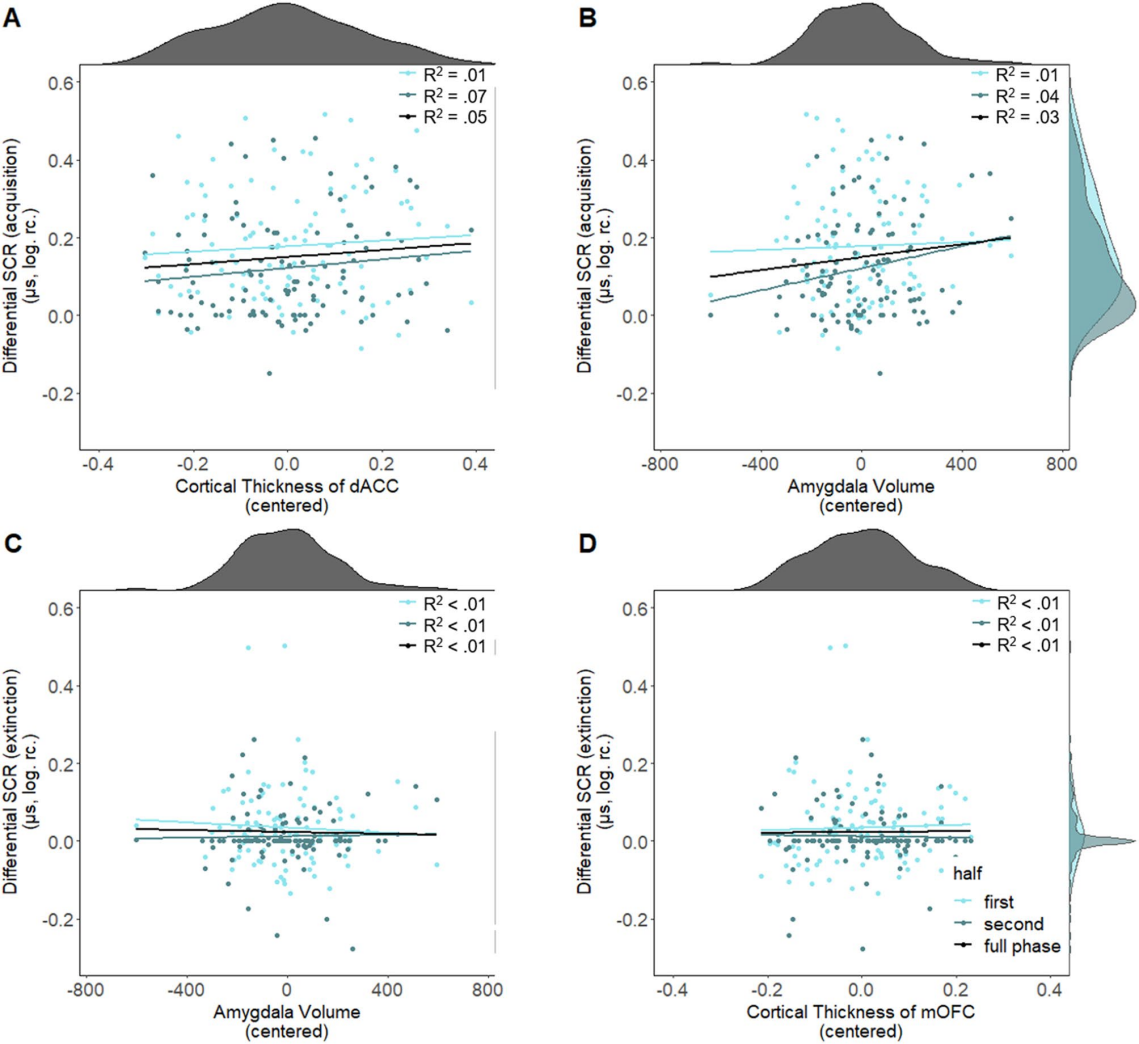
Scatterplots with marginal densities illustrating the (absence of) associations between average differential SCR [(CS+) − (CS−)] during acquisition training (illustrated also for the first and second half of acquisition separately) and (**A**) cortical thickness of the dACC and (**B**) amygdala volume as well as between differential SCR [(CS+) − (CS−)] during extinction (illustrated also for the first and second half of acquisition separately) and (**C**) amygdala volume as well as (**D**) cortical thickness of the mOFC. Data points are color-coded depending on the first half (light blue), second half (blue).

**Figure 6:**
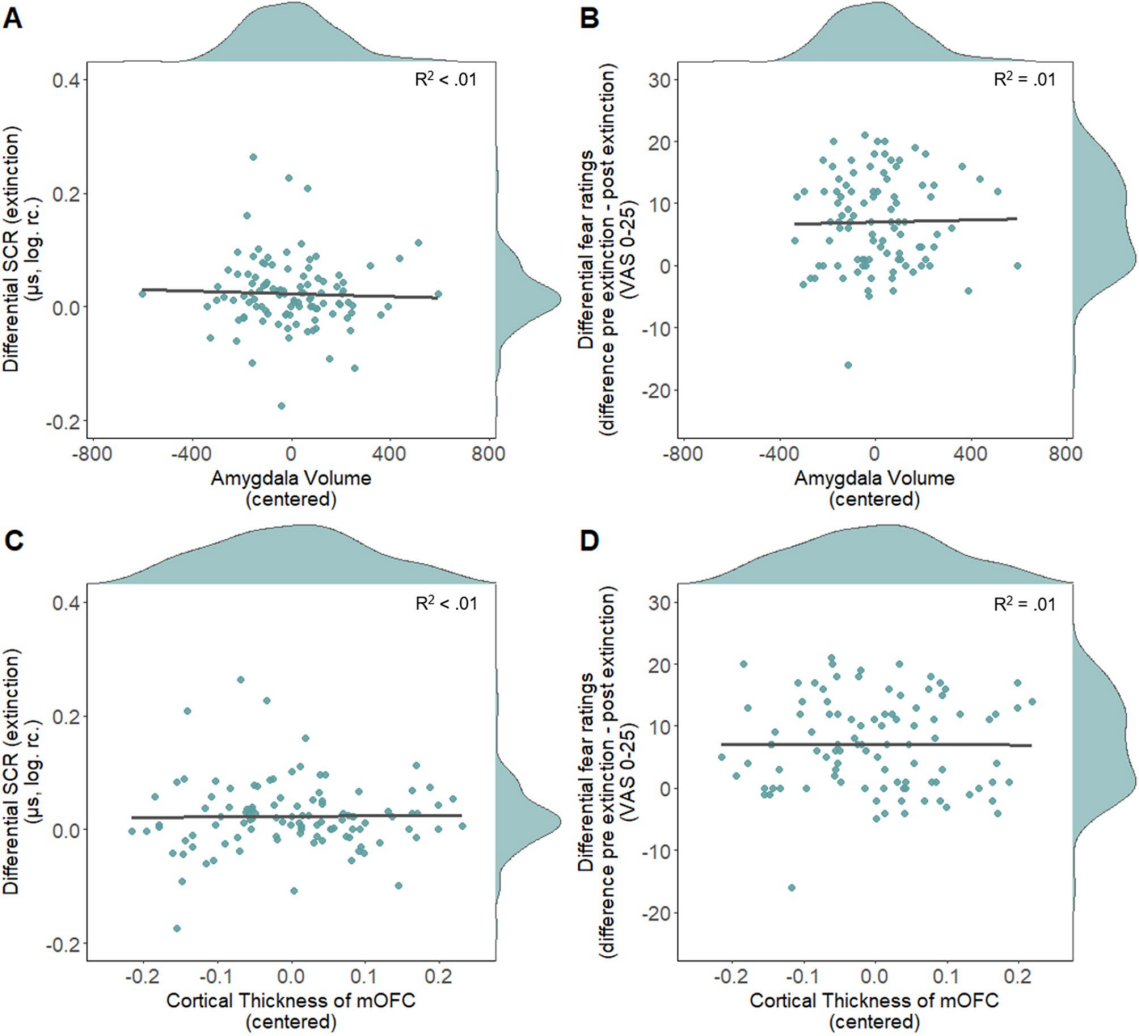
Scatterplots with marginal densities illustrating the (absence of) associations between average differential SCR [(CS+) − (CS−)] during extinction training and (**A**) amygdala volume and (**C**) cortical thickness of the mOFC and (**B**) (illustrated also for the first and second half of acquisition separately in Fig. 5) as well as between differential pre–post extinction fear ratings [[(CS+_pre_) − (CS−_pre_)] − [(CS+_post_) − (CS−_post_)] and (**B**) the amygdala and (**D**) the mOFC thickness.

**Figure 7:**
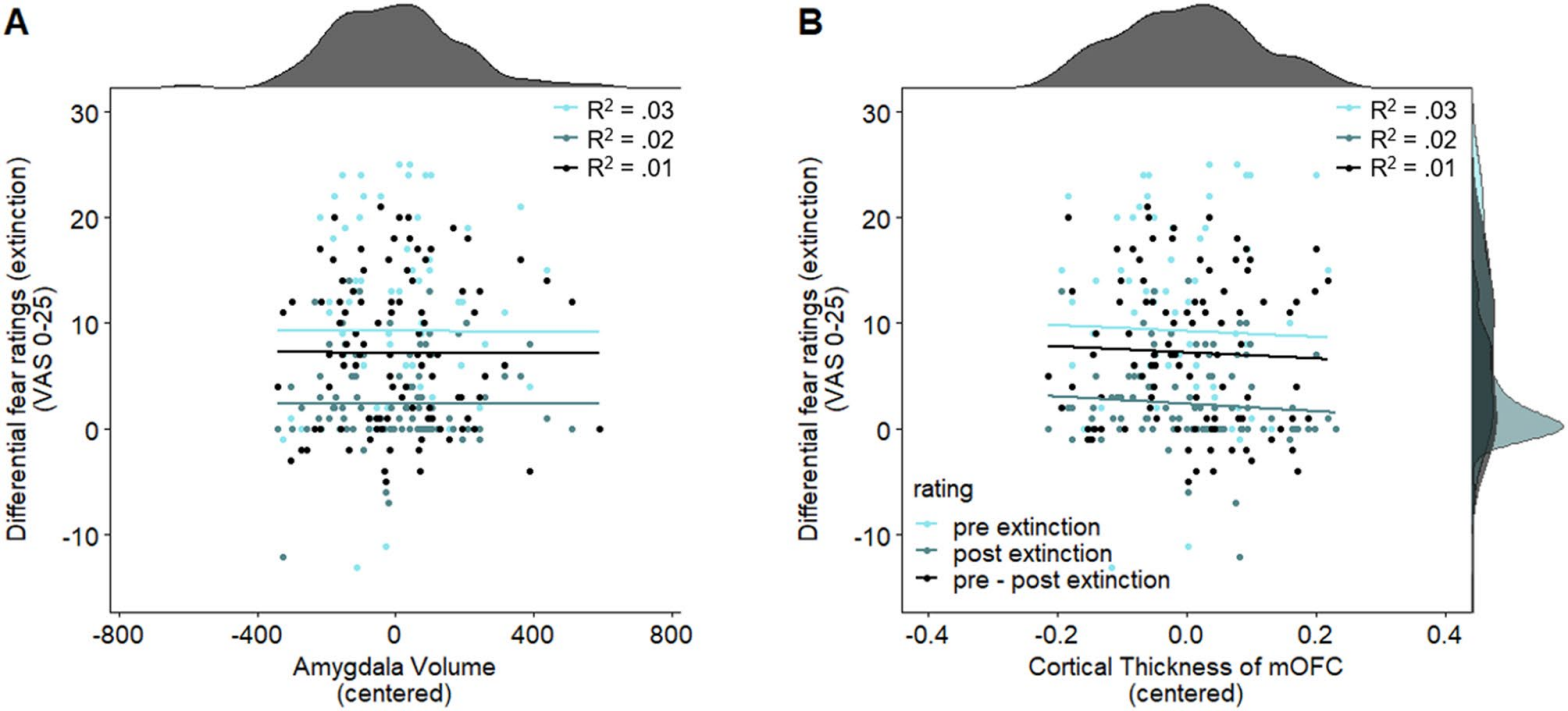
Scatterplots with marginal densities illustrating the (absence of) associations between differential pre, post and pre–post extinction fear ratings for (**A**) amygdala and (**B**) mOFC. Data points are color-coded to show fear ratings pre (light blue) and post (blue) extinction as well as the pre–post extinction difference score [[(CS+_pre_) − (CS−_pre_)] − [(CS+_post_) − (CS−_post_)] (black).

Our analyses did not replicate previous reports of a significant positive association between the cortical thickness of the dACC and subcortical volume of the amygdala during fear acquisition training as assessed through mean differential SCR and post-acquisition differential fear ratings (see Fig. 4). This was true either when considering the full acquisition phase or the first and second half of acquisition training separately (see Fig. 5A,B; Table 2). Similarly, our additional non-preregistered analyses aiming to replicate previous findings did not provide evidence for a significant association between SCR to the CS+ and CS− separately and thickness of the dACC (see Supplementary Material Section 3.1) or between differential SCR or post-acquisition fear ratings and thickness of the insula (see Supplementary Material Section 3.2). Likewise, no significant association was observed between amygdala volume or mOFC thickness and differential SCR (full phase, first and second half, see Figs. 5C,D, 6A,C) as well as differential ratings during extinction ([pre-post extinction ratings], pre ratings, post ratings, see Figs. 6B,D, 7) see Table 2). For robustness, we checked whether the exclusion of outliers (> 3 SD below or above mean), in fear ratings or SCR affects the results. The analyses were rerun after excluding one participant based on post-acquisition fear ratings, one based on pre-post extinction fear ratings and four based on differential SCR during extinction. The pattern of results remains comparable after excluding outliers for the respective analyses, i.e. all results remained non-significant. For full results see Supplementary Table 4.

**Table 2:** Results of regression analyses with cortical thickness/subcortical volume and differential SCR and fear ratings during fear acquisition and extinction training (controlled for sex and TIV) and Bayes factor BF_01_ providing relative evidence for the intercept-only against the hypothesis based regression model. Bold values indicate pre-registered hypotheses.

	dACC		Amygdala		mOFC	
	Regression	BF_01_	Regression	BF_01_	Regression	BF_01_
**Fear acquisition training**						
Differential SCR: full phase	***F*(3,103) = 1.93**, ***p* = 0.13 *R***^**2**^** = 0.05**	5.00	***F*(3,103) = 0.93**, ***p* = 0.43,** ***R***^**2**^** = 0.03**	18.18	–	–
Differential SCR: first half	***F*(3,103) = 0.50**, ***p*** **= 0.68,** ***R***^**2**^** = 0.01**	32.26	***F*(3,103) = 0.22**, ***p*** **= 0.89,** ***R***^**2**^** = 0.01**	47.62	–	–
Differential SCR: second half	***F*(3,103) = 2.66**, ***p*** **= 0.052,** ***R***^**2**^** = 0.07**	1.99	***F*(3,103) = 1.61**, ***p*** **= 0.19,** ***R***^**2**^** = 0.04**	7.52	–	–
Differential post acquisition fear ratings	***F*(3,99) = 2.49**, ***p*** **= 0.06,** ***R***^**2**^** = 0.07**	3.01	***F*(3,99) = 1.51**, ***p*** **= 0.22,** ***R***^**2**^** = 0.04**	11.24	–	–
**Extinction training**						
Differential SCR: full phase	–		***F*(3,103) = 0.05**, ***p*** **= 0.99,** ***R***^**2**^** < 0.01**	58.82	***F*(3,103) = 0.04**, ***p*** **= 0.99,** ***R***^**2**^** < 0.01**	58.82
Differential SCR: first half	–		*F*(3,103) = 0.15, *p* = 0.93, *R*^2^ < 0.001	52.63	***F*(3,103) = 0.08**, ***p* = 0.97,** ***R***^**2**^** < 0.01**	58.82
Differential SCR: second half	–		*F*(3,103) = 0.07, *p* = 0.98, *R*^2^ < 0.001	55.56	***F*(3,103) = 0.03**, ***p*** **= 0.99,** ***R***^**2**^** < 0.01**	62.50
Differential fear ratings [pre–post extinction]	–		***F*(3,93) = 0.23**, ***p*** **= 0.88,** ***R***^**2**^** = 0.01**	43.48	***F*(3,93) = 0.23**, ***p* = 0.88,** ***R***^**2**^** = 0.01**	45.45
Differential pre extinction fear ratings (fear recall)	–		*F*(3,94) = 0.89 *p* = 0.45, *R*^2^ = 0.03	20.41	*F*(3,94) = 0.89, *p* = 0.45, *R*^2^ = 0.03	21.28
Differential post extinction fear ratings	–		*F*(3,100) = 0.62 *p* = 0.60, *R*^2^ = 0.02	30.30	*F*(3,100) = 0.79, *p* = 0.50, *R*^2^ = 0.02	24.39

In addition to traditional null hypothesis significance testing (NHST), we computed Bayes factors in order to obtain relative evidence against a significant relationship between brain morphology and indices of fear learning. The calculated Bayes factors indicate moderate to strong evidence (BF_01_ > 3) for the null or intercept-only model. For one of the tested regression models, only weak evidence for the null model was found (BF_01_ = 1.99). Overall, these results demonstrate that there is little reason to believe that morphology in these regions is a significant predictor of conditioned responding during acquisition or extinction training in our study.

Robustness analyses considering data derived from the left and right hemisphere separately (Supplementary Section 2.1, Supplementary Table 1), without the pre-registered covariates (see Supplementary Material Section 2.2, Supplementary Table 2), with raw instead of log-transformed and range corrected SCR scores (see Supplementary Material Section 2.3, Supplementary Table 3) and after removing outliers (see Supplementary Material Section 2.4, Supplementary Table 4) yielded comparable results for both acquisition and extinction training.

### Contingency awareness does not moderate a putative association of dACC thickness and fear learning proxies

Analyses did not confirm our pre-registered exploratory hypothesis of a significant moderation of a putative association between dACC thickness and fear learning proxies (differential SCR and differential subjective fear ratings) during fear acquisition training by contingency awareness (aware, unaware, uncertain). The analysis unsurprisingly revealed that awareness is a significant predictor for differential SCR and fear ratings during fear acquisition training (SCR: *β* = 0.08, *p* = 0.01, ratings: *β* = 4.45, *p* = 0.02) which, however, does not moderate the putative relationship between dACC thickness and fear learning proxies (for full results see Supplementary Material Section 1.1).

### Aiming to replicate previous associations between amygdala volume and trait anxiety

Non-preregistered analyses in the current sample did not replicate previous reports of a significant association between amygdala volume—considering averaged values as well as left and right hemisphere separately—and trait or state anxiety as measured prior to acquisition training or prior to extinction training (for full results see Supplementary Material Section 3.3).

## Discussion

Research regarding a potential association between physiological and subjective measures of conditioned responding during acquisition and/or extinction and its retention and inter-individual differences in brain morphology is sparse to date and most of the few inconsistent results originate from early studies in extremely small samples.

Here, we attempted to (conceptually) replicate these previous findings in a large sample. Our results do not provide support for structural brain-behavior relationships during fear acquisition training and extinction. More precisely, we did not replicate previously reported significant associations between differential SCR or fear ratings and cortical thickness of the dACC, the insula or volume of the amygdala during fear acquisition training or between amygdala volume and mOFC thickness during extinction. Bayes factors provide moderate to strong evidence against a relationship between brain morphology in these regions and physiological or subjective measures of conditioned responding during acquisition or extinction training. Yet, it should be acknowledged that we do not provide a formal close or direct replication as we tested these previously reported associations in a fear conditioning paradigm in which procedural features differ from those in previous work in several ways (i.e., conceptual replication) (see Table 1): the reinforcement ratio, the number of trials, immediate vs. delayed extinction as well as measurement procedures to quantify SCR and estimates of brain morphology.

In more detail, the current study employed a 100% reinforcement rate while many previous studies used partial reinforcement (17–80%)^14–17,20^ and only three studies also employed a 100% reinforcement rate^20,22,23^. The probability with which the CS+ is coupled with the US during fear acquisition training contributes to the speed of fear acquisition and subsequent extinction learning, with partial reinforcement slowing both the development of conditioned responding and extinction learning^4,31,32^. It has also been argued that partial reinforcement rate may promote the manifestation of individual differences as opposed to the ‘strong experimental situation’ induced by 100% reinforcement rate^32^, but this ideas still needs to be tested empirically.

Also the number of trials included in the experimental phases differs substantially among previous work (range acquisition: 5–36 trials; range extinction: 8–18 trials per CS type, see Table 1) which renders classifications into ‘first half’ and ‘second half’ inherently ambiguous and difficult to interpret across studies without considering procedural specifications.

In the current study, we aimed to replicate a positive association between amygdala volume and differential SCR observed during the first but not the second half of acquisition training^14^. Critically however, Cacciaglia et al.^14^ presented a total of 36 trials per CS type during acquisition training (i.e., 18 CS+ and CS− during both the first and second half of acquisition training), while the current study design included a total of 14 CS+ and CS− trials during acquisition training. Consequently, the total number of trials during fear acquisition training in the present study was shorter than the first half of the previous study. We did, however, employ 100% compared to 50% reinforcement rate^14^, which likely led to faster fear acquisition in our study. Yet, we did not observe a significant association between amygdala volume and differential SCR or differential post-acquisition ratings when considering the full acquisition training phase—largely overlapping with the first half of acquisition training in^14^—or the first or second half of our acquisition training phase (6 and 7 trials per CS type respectively).

Another important difference that should be acknowledged when interpreting the current results is whether extinction took place immediately after fear acquisition training (i.e., immediate extinction) or after a time delay (i.e., delayed extinction) such as 24 h. Previous studies reporting a relationship between prefrontal thickness and fear learning proxies during extinction learning^14,16^, extinction recall^20,25^ or renewal^24^ have all employed an immediate extinction paradigm, while our own data as well as those by Hartley et al.^20^ (sample 2) are based on a 24 h-delayed extinction procedure.

None of the studies considered here employed fear learning paradigms explicitly instructing the CS–US relationship^14–16,20,21,24,25^, but reinforcement rate is another factor known to influence CS–US contingency awareness^2,4,33^. Thus, we explored whether the previously reported relationship between fear learning proxies and brain morphology is masked by a modulation by contingency awareness. Our results, however, show that awareness affects differential SCR during fear acquisition training, but does not modulate a hypothesized brain behavior relationship. It should be noted though that the group of participants who were unaware was very small (N = 7) and hence this putative null finding needs to be interpreted with caution.

Besides differences in the experimental paradigms across studies, methods for measuring cortical thickness and subcortical volumes as well as SCR quantification differed between previous studies as well as previous work and our work. Assessment of cortical thickness and brain volume was nearly exclusively performed through automated methods as implemented in the software Freesurfer^16,17,20,21,24,25^ while only a single study employed manual segmentation of subcortical structures^14^. It can be speculated that employing different methods to assess brain morphology might have contributed to the non-identical results obtained in two previous studies which were based on largely overlapping samples^14,16^. In contrast to the brain morphometry analyses, a plethora of different SCR quantification approaches was employed in previous work, most of which differed from our approach (see Table 1).

In sum, experimental paradigms and methodological procedures differ substantially between the studies in the field including ours. Yet, “It is tempting to explain away nonsignificant results in a line of studies by minor differences in the method, even when random variation is a much more likely explanation.” cf.^34^—in particular in small, sub optimally powered studies which represent the major share of the work we based our hypotheses on.

While we were not able to (conceptually) replicate any of the previously reported structural brain behavior associations in a large sample of healthy adults, general functional brain activation patterns during fear acquisition and extinction training are relatively well established^18,19^. Of note, functional activation patterns during fear acquisition, extinction or retention of extinction^18,19^ involve all brain regions that have been reported previously to show structural brain-behavior associations during fear conditioning studies^14–17,20,21,24,25^. However, it is unclear whether and how inter-individual differences in structural characteristics relate to inter-individual differences in functional activation during different phases of a fear conditioning paradigm (as discussed by^16^). Furthermore, it has been suggested that brain structure and function may not be uniformly related but may show high coupling in sensory areas and particularly low coupling in the default mode or salience network^35^. Critically, the so-called salience network comprises the dACC, orbital frontoinsular as well as limbic regions such as the amygdala^36^—all regions functionally related to fear processing^18,19^ and serving as regions of interest in this study. In sum, while functional brain activation patterns underlying fear conditioning are well established, associations with brain morphology in the same regions seem questionable. One possible reason could be the low association of brain structure and function especially in neural circuitry underlying fear learning. Moreover, a recent systematic attempt to replicate a number of reported associations between cortical thickness or grey matter volume and psychometric variables and psychological measurements in a large sample of healthy adults showed no significant associations in more than 90% of the performed analyses^5,13^. This led the authors to conclude that such associations are unlikely to be found and that—even with identical experimental designs—it is highly unlikely to replicate associations between brain morphology and psychometric variables. Importantly, replication rates decreased with decreasing sample size of the replication sample^5^ and associations have been shown to stabilize and become more reproducible in very large samples with N = ~  2000^37^. It is well recognized that also in initial studies, small sample sizes are generally linked to low statistical power and inflated effect sizes. Low statistical power does, however, not only reduce the likelihood to detect a true effect but also reduces the likelihood with which a significant finding actually reflects a true population effect. Consequently, small sample sizes are assumed to lead to low replication rates, as discussed for task-based fMRI^27,38^. In light of this, it is maybe not surprising that we were unable to (conceptually) replicate previous findings which are often derived from extremely small sample sizes with 10–14 participants^20,21,24,25^. Yet, we were also unable to (conceptually) replicate findings derived from (somewhat) larger samples^16,17^.

While structural MRI measures themselves have been shown to have excellent test–retest reliability^28,29^, the reliability of measures of defensive responding, such as SCR and fear ratings during fear acquisition and extinction training remains understudied and underreported. While within-subject reproducibility and test–retest reliability has been established with intermediate reliability coefficients for conditioned SCR across time intervals ranging from 3 weeks to 8 months (8 months^39^, 3 weeks^40^, 8–12 weeks^41^) for maximum CS+ responding, CS− responding and CS+/CS− discrimination in SCR^39–41^. Reliability of other measures of defensive responding should also be systematically investigated in order to draw conclusions about potential reasons for the lack of associations presented here. This is important, as measurement reliability puts an upper bound to the maximum correlation that can be observed^42^ and it is likely that early reports of correlation coefficients as high as 0.8 (see Fig. 1A) might be inflated and implausibly high.

In conclusion, in line with recent studies questioning the existence and robustness of structural brain-behavior associations in healthy adults, we did not observe any associations between cortical thickness or subcortical volume in a number of brain regions and differential SCR and fear ratings as proxies for the acquisition and extinction of conditioned fear. Yet, if a finding cannot be replicated conceptually this may indicate that the association may only be observable under very specific boundary conditions. If true, this hampers the generalizability of the findings substantially. It is important to point out, however, that our work cannot be taken as evidence against the findings reported previously for several reasons: First, we do not provide a close or direct replication of any of these previous studies and second, the absence of a significant p-value in our study and the presence of a significant p-value in a given previous study cannot be taken to infer non-replication of an effect in absence of a formal statistical evaluation of replication (see^43^ for a formal framework on replicability). Nevertheless, the current results cast some doubt on the idea that differences in brain morphology are likely to contribute to inter-individual differences in fear learning processes.

Future studies should employ longitudinal designs in order to investigate whether changes in brain morphology over time or measures of structural connectivity may have greater predictability for inter-individual differences in defensive responding. Most importantly, however, a focus on measures in general^26,44^ and the reliability of the measures used in studies on inter-individual differences in conditioned responding as well as structural and functional brain imaging are key and need to be scrutinized. In fact, the best research idea and the most transparent reporting methods cannot make up for inappropriate and/or unreliable measures employed. It may be time to take a step back and focus more on our measures because the reliable and reproducible quantification of measurements is fundamental to research in general and individual difference research in particular.

## Methods and materials

### Participants

The data set is part of the baseline measurement of a longitudinal fear conditioning study. For the current study, fear ratings, SCR and structural neuroimaging data from the first test-timepoint (T0) which consisted of two experimental days (Day 1: habituation, acquisition, Day 2: extinction) were included whereas reinstatement test (Day 2) and fMRI data were not analyzed here. All methods were carried out in accordance with relevant ethical guidelines and regulations. All experimental protocols were approved by the local ethics committee (PV 5157, Ethics Committee of the General Medical Council Hamburg). All participants gave written informed consent before participation. The current data set as well as the analysis code are made publicly available (10.17605/osf.io/y2jv9). The data set has also been used as a case example in our previous publication on a methodological question different from the question addressed here^45^. As pre-registered, several participants had to be excluded from the initial sample (N = 120) due to the following reasons: For Day 1, one participant had to be excluded due to missing data, three participants due to non-responding (no SCR response to the US on more than 9 out of 14 occasions) and an additional participant due to a deviating protocol on both days. Moreover, one participants was excluded due to technical issues on Day 2 in addition to five participants due to non-responding in SCR on Day 2 (see ‘Physiological Measurements—SCR’ for definition). Two participants were, as pre-registered, excluded from the analysis due to assumed technical issues on Day 2. Only after the data analysis did we realize that data for these participants was complete for fear acquisition and extinction training as technical issues only occurred in the subsequent reinstatement phase. Hence, these two participants could have been included but were excluded as pre-registered. After exclusions, structural and psychophysiological data of N = 107 participants (71 females, mean ± SD age of 24.4 ± 3.7 years, age range 18–34, state-trait anxiety inventory (STAI)^46^ mean ± SD of 34.6 ± 7.2, range of 24–55) were included in the analyses. Due to missing data in fear ratings from fear acquisition training, N = 103 participants (67 females) were included into the analyses of fear ratings. Fear ratings pre extinction are missing from nine and ratings post extinction are missing from three participants, resulting in a total of N = 95 participants (64 females) for the comparison of pre and post extinction ratings.

### Stimuli

An electrotactile stimulus administered to the back of the participant’s right hand served as the US. The stimulus comprised three 2 ms electrotactile rectangular pulses with an interpulse interval of 50 ms delivered 200 ms before CS+ offset. The pulse was generated by a Digitimer DS7A constant current stimulator (Welwyn Garden City, Hertfordshire, UK) and delivered through a 1 cm diameter platinum pin surface electrode (Specialty Developments, Bexley, UK) placed between the metacarpal bones of the index and middle finger. US intensity was individually calibrated in a step-wise procedure to reach an unpleasant, but not painful level for each participant.

Two light grey fractals served as conditioned stimuli, the allocation of which to CS+ and CS− as well as the order was counterbalanced across participants. All stimuli were presented on grey background.

### Experimental design

Participants completed a two-day paradigm consisting of habituation and acquisition training on Day 1 and extinction training, reinstatement administration and reinstatement test on Day 2. In the current study, only data from acquisition and extinction training are presented. For both acquisition and extinction training, CS+ and CS− were each presented 14 times in pseudo-randomized order for a duration of 6–8 s (mean duration: 7 s). Inter-trial intervals (ITIs) consisted of a white fixation cross presented for 10–16 s (mean duration: 13 s). Presentation of all stimuli on a grey background and stimulus timing were controlled by Presentation software (Version 14.8, Neurobehavioral Systems, Inc, Albany California, USA).

### Fear ratings and contingency awareness

Fear ratings were completed after habituation and acquisition training on Day 1 as well as before extinction training and after reinstatement on Day 2. Participants were asked how much ‘stress, fear and tension’ they experienced when they last saw the CS+ and CS−. The ratings after reinstatement test referred to the first CS presentation per CS type after reinstatement administration and the last presentation during the test phase respectively (note that this phase was, however, not analyzed here). Answers were given within a 5 s time window on a visual analog scale (VAS) ranging from zero (answer = none) to 100 (answer = maximum), re-scaled to 0–25. A standardized post-experimental awareness interview adapted from^47^ was conducted after acquisition training in order to assess CS–US contingency awareness. Subsequently, participants were classified as aware, unaware or uncertain of CS–US contingencies by the experimenter.

### Physiological measurements—SCR

Physiological data were recorded with a Biopac MP100-amplifier system (BIOPAC Systems Inc, Goleta, California, USA) and AcqKnowledge 3.9.2 software and converted from analog to digital using a CED2502-SA with Spike 2 software (Cambridge Electronic Design, Cambridge, UK). Skin conductance response was measured by placing two self-adhesive, hydrogel Ag/AgCl electrodes on the distal and proximal hypothenar on the palmar side of the left hand. Data was continuously recorded at 1000 Hz with a gain of 5 or 10 µΩ and down-sampled to 10 Hz.

In line with previous recommendations^48,49^, data were scored semi-manually as a trough to peak (TTP) response between 0.9 and 3.5 s after CS onset using the custom-made program EDA view (developed by Prof. Dr. Matthias Gamer, University of Würzburg). Rise time was set to a maximum of 5 s. Each scored SCR was checked visually, and the scoring suggested by EDA View was corrected if necessary. For example, the algorithm sometimes suggested an SCR outside the scoring window or the foot or trough were misclassified especially when several responses overlapped. Data with recording artifacts or excessive baseline activity (more than half of the response amplitude) were scored as missing values and excluded from the analysis. Response increases smaller than 0.01 μS in the pre-defined time window were set to zero, for a justification see^45^. Raw SCR amplitudes were log transformed for purposes of normalization and range corrected by dividing each SCR by the maximum SCR (to CS or US) for each participant and day.

Physiological ‘non-responding’ on Day 1 was defined as no SCR response to the US on more than 9 out of 14 occasions. On Day 2, ‘non-responding’ was defined as no SCR response to any of three USs during reinstatement. A total of eight participants was classified as ‘non-responders’^45^.

### MRI data acquisition and analysis

T1-weighted structural images (1 × 1 × 1 mm) were acquired on Day 2 with a 3T PRISMA whole body scanner (Siemens Medical Solutions, Erlangen, Germany) using a 64-channel head coil and magnetization prepared rapid gradient echo (MPRAGE) sequence (TR = 2300 ms, TE = 2.98 ms, field of view = 192 × 256 mm, 240 slices).

Cortical thickness and volume of subcortical brain regions were reconstructed using the brain imaging software Freesurfer 6.0.1^7–9^. Thus, the regions of interest used in the current study are defined based on the areas implemented in Freesurfer and visualizations can be found online (https://surfer.nmr.mgh.harvard.edu/). The surface-based stream that yields measures of cortical thickness includes an initial Talairach registration, bias field correction, skull stripping, white matter classification, surface generation and gyral labeling^7^. Similarly, the volume-based or subcortical stream involves an initial Talairach registration, initial volumetric labeling, bias field correction, nonlinear volumetric atlas registration and volumetric labeling of subcortical structures^50^. Cortical parcellation is based on the Desikan-Killiany cortical atlas^51^ implemented in Freesurfer.

### Statistical analysis

The success of fear acquisition and extinction training was assessed by performing *t* tests and ANOVAs comparing averaged SCR elicited by the CS+ and CS− during acquisition and extinction training and fear ratings to the CS+ and CS− after acquisition as well as before and after extinction training. The SCR to the first CS+ and CS− of acquisition training were excluded from all analyses as no learning could possibly have taken place as the first CS+ presentation and the corresponding SCR occur prior to the first US presentation. Paired samples *t* tests were conducted to test for significant differences in cortical thickness and subcortical volume between the left and right hemisphere for the dACC, mOFC, insula and amygdala. For all other analyses, volumina of both hemispheres of a region were averaged and, as pre-registered, sex and total intracranial volume (TIV) were included as covariates.

To test the hypothesis that dACC thickness and amygdala volume predict conditioned responding during acquisition training, separate linear regressions predicting average differential [(CS+) – (CS−)] SCR during acquisition training and differential [(CS+) – (CS−)] post-acquisition fear ratings from dACC thickness and amygdala volume were conducted. Please note that the pre-registration used an ambiguous formulation regarding the ratings. We had used the term “mean differential fear rating” but there was only one rating after the acquisition phase. Additional analyses used average SCR responding during the first half (i.e. trials two to seven for acquisition training and trials one to seven for extinction) and second half (i.e., trials eight to fourteen for acquisition training and extinction) of acquisition and extinction training (pre-registered for amygdala, also performed for dACC for completeness).

For extinction, equivalent analyses were set up with average differential [(CS+) – (CS−)] SCR across all trials during extinction learning and fear ratings as outcome variables and amygdala volume and mOFC thickness as predictors. Regarding the fear ratings, our pre-registration used an ambiguous formulation (“mean differential fear ratings”). As we, in contrast to SCR, did only assess ratings prior to and after but not during extinction training, we specify here that we used the difference in ratings pre and post extinction [pre extinction − post extinction]. For completeness, exploratory analyses were also performed with pre and post extinction ratings instead of the difference score. As pre-registered, mOFC thickness was also tested as a predictor for average differential SCR during first and second half of extinction.

Pre-registered exploratory moderated regression analyses were conducted with dACC as predictor, averaged differential [(CS+) − (CS−)] SCR during fear acquisition training and differential [(CS+) − (CS−)] fear ratings after acquisition training as outcome and contingency awareness as moderator variables (reported in the Supplementary Material Section 1.1, Supplementary Figure 1).

Additionally, some non-preregistered analyses were performed for completeness, as additional robustness checks to the main analyses (because significant differences between volumina/thickness emerged between both hemispheres) and in order to replicate specific findings from individual studies^16,20,21^. The results of these analyses can be found in the Supplementary Material.

1. The regression analyses testing for the main pre-registered hypotheses were also performed separately for left and right hemisphere. Full results are reported in the Supplementary Material (see Section 2.1 and Supplementary Figures 2 and 3 as well as Supplementary Table 1).
2. Robustness analyses were performed for all main pre-registered analyses including sex as covariate and no covariates in order to ensure that the current results can be generalized to different combinations of covariates^52^. Model fit comparisons were further performed in order to show whether including covariates added predictive power. Full results can be found in the Supplementary Material (see Section 2.2 and Supplementary Table 2).
3. As Milad et al.^21^ reported a correlation of cortical thickness of the dACC with SCR to CS+ and CS− only, we performed correlations with dACC thickness and CS+ and CS− elicited SCR. Additionally, we computed partial correlations controlling for sex and TIV. Results are reported in the Supplementary Material (see Section 3.1, Supplementary Figure 4 and Supplementary Table 5).
4. As Hartley et al.^20^ reported an association between the right posterior insula and CS+/CS− discrimination during acquisition training, we conducted a correlational analysis for left, right and average insula thickness and differential SCR and fear ratings during acquisition training. Results are reported in the Supplementary Material (see Section 3.2, Supplementary Figure 5 and Supplementary Table 6).
5. As some^53,54^ but not all^16^ previous studies reported an association between trait anxiety and amygdala volume, partial correlations were calculated in order to test for a relationship between trait anxiety as well as state anxiety prior to acquisition and extinction training and amygdala volume in addition to amygdala volume and state anxiety one day after acquisition training. Results are reported in the Supplementary Material (see Section 3.3, Supplementary Figure 6 and Supplementary Table 7).

In addition to traditional null hypothesis significance testing (NHST), we computed Bayes factors for all analyses, allowing us to not only to find evidence for our tested hypotheses but to quantify the evidence in favor of the null hypotheses. In the current study, we used the R package “BayesFactor”^55^ in order to calculate Bayes factors to obtain relative evidence for the tested regression (or correlation) model against a null or intercept-only model. Here, we report the Bayes Factor BF_01_ to directly show how much more likely the null hypothesis is relative to the alternative hypothesis given the data. Bayes factors (BF_01_) > 1 are generally considered as evidence *against* the alternative hypothesis or *for* the null hypothesis^56^. More specifically, weak evidence for the null hypothesis is defined as BF_01_ = 1–3, moderate evidence as BF_01_ = 3–20 and strong evidence as BF_01_ = 20–150^57^.

All analyses and data visualizations were performed with the Software package R (Version 1.2.5033) using the following packages: ggpubr^58^, ggplot2^59^, cowplot^60^, writexl^61^, car^62^, jtools^63^, readr^64^, broom^65^, ggfortify^66^, tidyr^67^, scales^68^, plyr^69^, RColorBrewer^70^, reshape2^71^, tidyverse^72^, grid^73^, gridExtra^74^, ggExtra^75^, patchwork^76^, apaTables^77^, MBESS^78^, egg^79^, ggm^80^, effectsize^81^, ppcor^82^, GGally^83^, psychReport^84^, lsr^85^, ez^86^, lattice^87^, dplyr^88^, rmarkdown^89^, Rmisc^90^, gghalves^91^, BayesFactor^55^. Power curves were plotted using open code https://www.statmethods.net/stats/power.html. Predictors for all linear regressions were centered in order to be able to investigate interactions and for easier interpretability. All effects are reported at significant level *p* < 0.05 unless indicated otherwise. Effect sizes are reported as Cohen’s *d*. No follow-up analyses were conducted since the pre-registered analyses did not yield any significant results.

## Supplementary information

Supplementary Information.
