# Supplementary material for: Revisiting potential associations between brain morphology, fear acquisition and extinction through new data and a literature review
Source: Sci Rep. 2020 Nov 16;10:19894. doi: 10.1038/s41598-020-76683-1 (PMC7670460; doi:10.1038/s41598-020-76683-1)
Supplement: Supplementary file 1 — Supplementary Information. [file 41598_2020_76683_MOESM1_ESM.pdf]

Supplementary Material

Revisiting potential associations between brain morphology, fear acquisition and extinction through new data and a literature review

Mana R. Ehlers[1], Janne Nold[1], Manuel Kuhn[1,2], Maren Klingelhöfer-Jens, Tina B. Lonsdorf[1]

[1] Department of Systems Neuroscience, University Medical Center Hamburg-Eppendorf, Martinistrasse 52, 20246 Hamburg

[2] Department of Psychiatry, Harvard Medical School, and Center for Depression, Anxiety and Stress Research, McLean Hospital, Belmont, MA 02478 USA.

Table of Contents

1. PRE-REGISTERED EXPLORATORY ANALYSES 2

1.1 Contingency awareness does not moderate the association between dACC thickness and defensive responding during fear acquisition training 2

2. NON-PRE-REGISTERED ROBUSTNESS ANALYSES 3

2.1 Robustness analyses considering data from right and left hemisphere separately 3

2.2 Robustness analyses including no covariates 7

2.3 Robustness analyses with raw SCR 9

2.4 Robustness analyses – outlier removed 10

3. ADDITIONAL, NON-PRE-REGISTERED ANALYSES AIMING TO (CONCEPTUALLY) REPLICATE PREVIOUSLY REPORTED FINDINGS 11

3.1 No association of dACC cortical thickness and SCR to the CS+ and CS- during acquisition training 11

3.2 No association between thickness of the insula and differential SCR and ratings during fear acquisition and extinction 12

3.3 No association of amygdala volume with trait and state anxiety 14

1. Pre-registered exploratory analyses

1.1 Contingency awareness does not moderate the association between dACC thickness and defensive responding during fear acquisition training

Contingency awareness has been identified as one factor contributing to inter-individual differences in defensive responding during fear acquisition training (Mertens & Engelhard, 2020; Tabbert et al., 2011). Here, we wanted to explore the pre-registered hypothesis that a potential association between dACC thickness and differential SCR and differential fear ratings during acquisition training might be moderated by contingency awareness.

A regression analysis with dACC thickness, contingency awareness as well as the pre-registered covariates sex and total intracranial volume (TIV) as predictors significantly predicted differential SCR ($F(4,101) = 3.52$, $p = .01$, $R^2 = .12$) and differential ratings ($F(4,97) = 3.65$, $p = .01$, $R^2 = .13$) during acquisition training. Adding the interaction term between contingency awareness and dACC thickness to the analysis still yielded a significant regression (SCR: $F(5,100) = 2.79$, $p = .02$, $R^2 = .12$, ratings: $F(5,96) = 3.24$, $p = .01$, $R^2 = .14$, see Supplementary Figure 1). The interaction between dACC thickness and contingency awareness was, however, not a significant predictor for differential SCR ($\beta = -.03$, $p = .86$) or ratings ($\beta = -15.86$, $p = .22$), rather the significant association was driven by awareness alone (SCR: $\beta = .08$, $p = .01$, ratings: $\beta = 4.45$, $p = .02$). These results should, however, be treated with caution since the group sizes differed substantially and were as low as 7 for the unaware group.

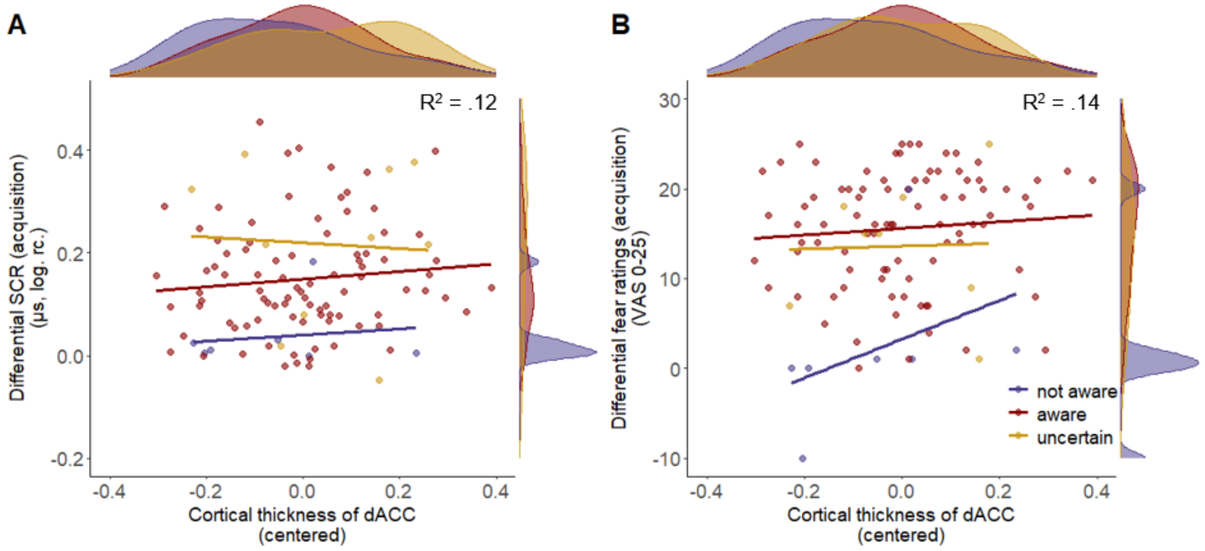

Supplementary Figure 1. Scatterplot with marginal densities illustrating the (absence of an) association between dACC thickness and (A) differential SCR and (B) differential fear ratings during acquisition training. Different awareness groups [“not aware” (N = 7), “aware” (N = 89) and “uncertain” (N = 10)] are color coded.

2. Non-pre-registered robustness analyses

For full transparency, we report robustness analyses to demonstrate that the results presented in the main manuscript (i.e., pre-registered analyses) are not contingent on specific analysis choices, such as using averaged values over both hemispheres (see 2.1), the choice of specific – albeit pre-registered – covariates (see 2.2), transformation of raw scores (see 2.3) or not removing outliers (see 2.4).

2.1 Robustness analyses considering data from right and left hemisphere separately

Previous research reported inconsistent lateralization (i.e., left or right lateralization) of the association of volume or cortical thickness and defensive responding during acquisition and extinction training. For instance, despite largely overlapping samples, Cacciaglia and colleagues observed a positive correlation between differential SCR and *left* amygdala volume, while Winkelmann and colleagues reported a positive correlation between differential SCR and *right* amygdala volume (Cacciaglia et al., 2014; Winkelmann et al., 2015). Further, effects were observed for right but not left insula and differential SCR during acquisition training (Hartley et al., 2011) and right but not left vmPFC and differential SCR during extinction training (Winkelmann et al., 2015). Hence, all major, preregistered analyses were also performed separately for left and right hemisphere for full transparency.

For that purpose, separate regression analyses with subcortical volume/cortical thickness derived from left and right hemisphere as predictor and differential SCR or differential fear ratings as outcome variables were performed for acquisition and extinction training for each brain region of interest (see methods, main manuscript). The pre-registered covariates sex and TIV were included as covariates for all analyses.

Similar to the results reported in the main manuscript, no significant association between any of the regions in any hemisphere was observed with either differential SCR or differential fear ratings during acquisition (see Supplementary Figure 2) or extinction training (see Supplementary Figure 3) (for full results see Supplementary Table 1) apart from a significant association between right dACC thickness and post-acquisition fear ratings ($F(3,99) = 2.73$, $p = .048$ $R^2 = .08$). However, the Bayes factor, $BF_{01} = 2.23$, for this association actually provides support for the null hypothesis (i.e. no significant relationship). Similarly, Bayes factors for all other analyses indicate that there is moderate to strong evidence ($BF_{01} > 3$) for the null or intercept-only model (see Supplementary Table 1).

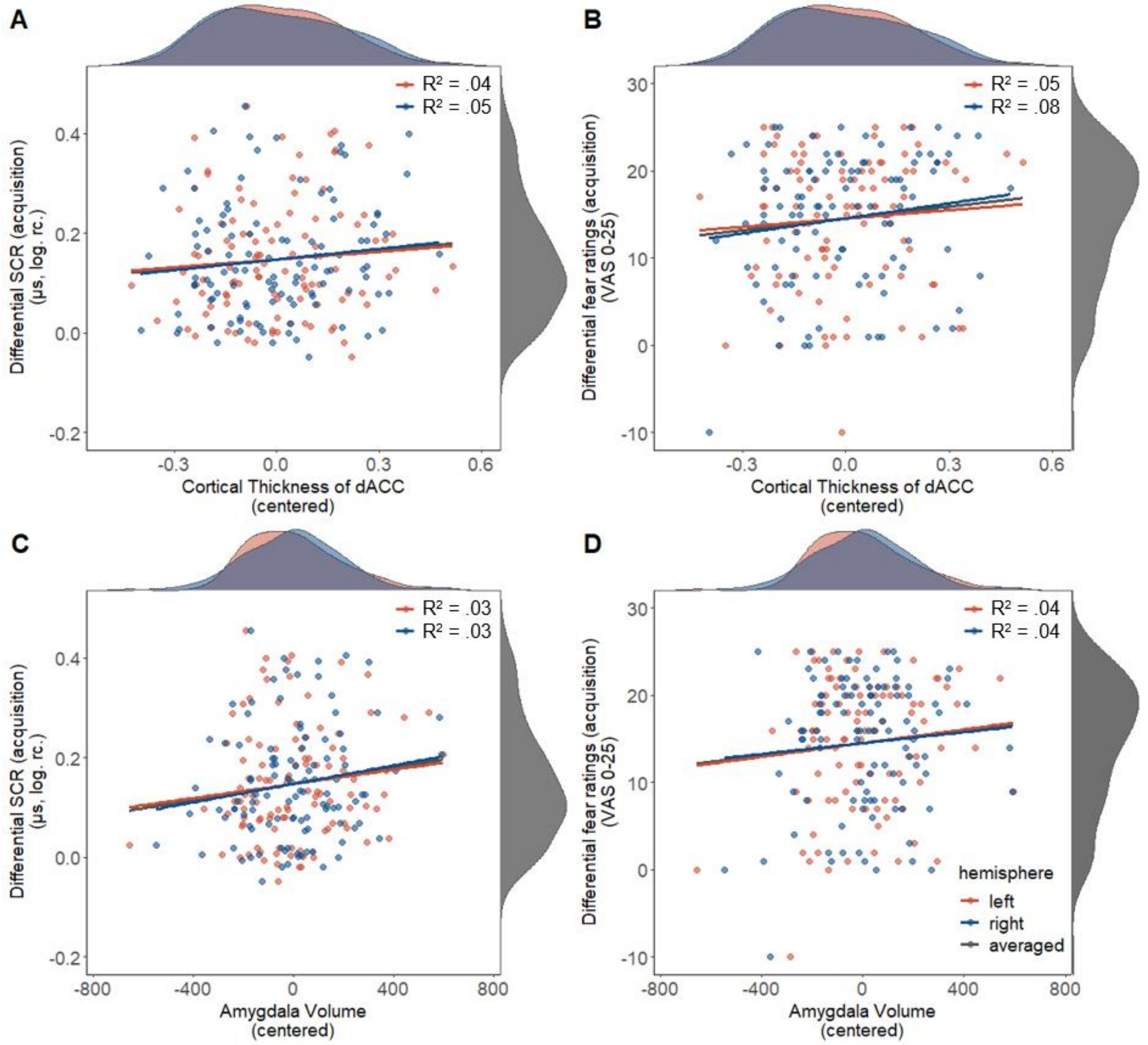

Supplementary Figure 2. Scatterplot and marginal densities illustrating the (absence of) associations between differential SCR during acquisition training with (A) dACC thickness and (B) amygdala volume as well as differential fear ratings post acquisition training with (C) dACC thickness and (D) amygdala volume. Data are illustrated separately for left (red) and right (blue) hemisphere. Regression lines are presented for both hemispheres (red and blue) as well as averages across both (dark grey).

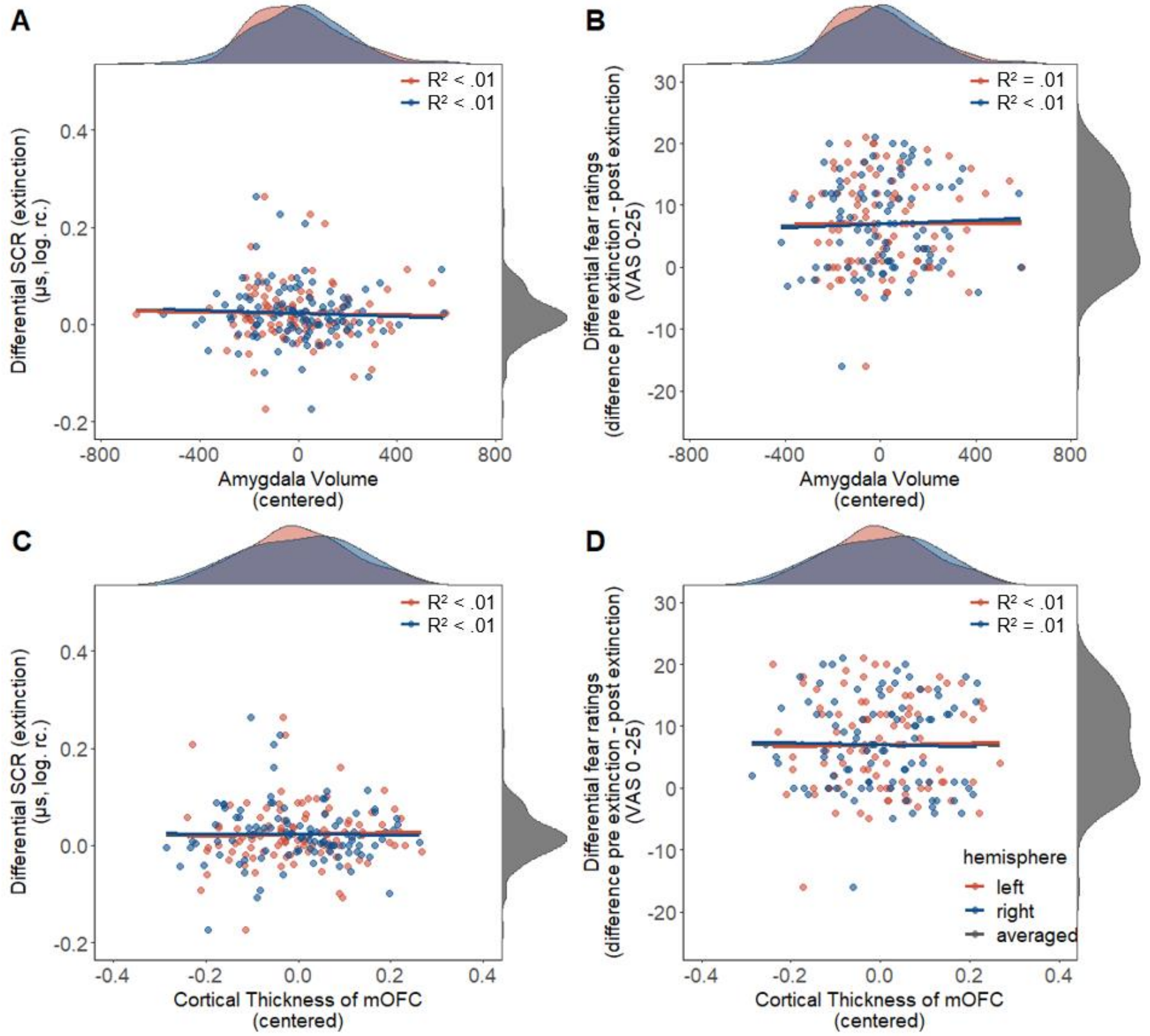

Supplementary Figure 3. Scatterplot and marginal densities illustrating the (absence of) associations of differential SCR during extinction training with (A) dACC thickness and (B) amygdala volume as well as of the difference between pre and post extinction differential ratings with (C) dACC thickness and (D) amygdala volume. Data are illustrated separately for left (red) and right (blue) hemisphere. Regression lines are presented for both hemispheres (red and blue) as well as averages across both (dark grey).

Supplementary Table 1. Results of regression analyses with left and right hemisphere cortical thickness/subcortical volume and differential SCR and fear ratings during fear acquisition and extinction training (controlled for sex and TIV) and Bayes factor BF_{01} providing relative evidence for the intercept-only model against the hypothesis based regression model.

**(A) Fear acquisition training**	**dACC**				**Amygdala**			
	**left**		**right**		**left**		**right**	
	**Regression**	**BF_{01}**	**Regression**	**BF_{01}**	**Regression**	**BF_{01}**	**Regression**	**BF_{01}**
Differential SCR	$F(1,103) = 1.28$, $p = .29$, $R^2 = .04$	11.90	$F(3,103) = 1.92$, $p = .13$, $R^2 = .05$	5.15	$F(3,103) = .88$, $p = .45$, $R^2 = .03$	19.23	$F(3,103) = .99$, $p = .40$, $R^2 = .03$	16.95
Differential post acquisition fear ratings	$F(3,99) = 1.74$, $p = .16$, $R^2 = .05$	7.94	**$F(3,99) = 2.73$, $p = .048$, $R^2 = .08$**	2.23	$F(3,99) = 1.50$, $p = .22$, $R^2 = .04$	11.11	$F(3,99) = 1.55$, $p = .21$, $R^2 = .04$	10.42
**(B) Extinction training**	**mOFC**				**Amygdala**			
	**left**		**right**		**left**		**right**	
	**Regression**	**BF_{01}**	**Regression**	**BF_{01}**	**Regression**	**BF_{01}**	**Regression**	**BF_{01}**
Differential SCR	$F(3,103) = .08$, $p = .97$, $R^2 < .01$	58.82	$F(3,103) = .06$, $p = .98$, $R^2 < .01$	58.82	$F(3,103) = .04$, $p = .99$, $R^2 < .01$	62.50	$F(3,103) = .09$, $p = .96$, $R^2 < .01$	52.63
Differential [pre – post extinction] fear ratings	$F(3,93) = .27$, $p = .85$, $R^2 < .01$	43.48	$F(3,93) = .24$, $p = .87$, $R^2 = .01$	45.45	$F(3,93) = .25$, $p = .86$, $R^2 = .01$	43.48	$F(3,93) = .27$, $p = .85$, $R^2 < .01$	41.67

2.2 Robustness analyses including no covariates

Our pre-registered analyses presented in the main manuscript included sex and TIV as covariates. It has been suggested to always include robustness analyses without covariates for full transparency (Simmons et al., 2011) and to ensure that presented results are not contingent on the covariates included.

Consequently, all main pre-registered analyses were also completed with either sex only or no covariates. In addition, for all analyses, the model fit of a regression with sex only or with sex and TIV as covariates was compared to a regression with morphometric estimates as the only predictor and no covariates. This serves the purpose to identify the best fitting model among those included and to determine whether the inclusion of specific covariates significantly alters model fit.

In brief, including only sex as covariate or no covariates yielded comparable results to those reported in the main manuscripts as no significant associations between brain morphology in any of the regions of interest and defensive responding in SCR and fear ratings during fear acquisition or extinction training were observed. Moreover, including covariates did not significantly improve model fit of the regression analyses.

More specifically, for acquisition training (for full results see Supplementary Table 2A), no significant associations between dACC thickness or amygdala volume and differential SCR or differential post acquisition fear ratings were observed with different combinations of covariates – with the exception of a significant association of amygdala volume and differential SCR during the second half of acquisition training when no covariates were included ($F(1,105) = 4.55$, $p = .04$, $R^2 = .04$) and Bayes factor of $BF_{01} = 0.65$ indicating moderate support for H_1. However, it should be noted that applying a simple Bonferroni correction for multiple comparisons would render this result no longer significant (i.e., correcting for 9 tests concerning the amygdala and SCR would result in an alpha level of $\alpha = .006$). Importantly, the regression model of interest only becomes significant when no covariates are included but not with any other combination of covariates further questioning the robustness of this single positive result.

Overall, model fit was not significantly improved by including covariates with the exception of including sex and TIV as covariates in the analysis of the relationship between dACC thickness and fear ratings ($F(1,99) = 5.38$, $p = .02$).

For extinction training, neither amygdala volume nor mOFC thickness could be significantly predicted from differential SCR or fear ratings regardless of the covariates included. In line with this, model fit was not significantly improved by the addition of covariates (for full results see Supplementary Table 2B).

Supplementary Table 2. Results of robustness analyses for morphology and indices of fear learning including different covariates

Structure	Outcome measure	Phase	Covariates: Sex		Covariates: None		Model fit compared to analysis with no covariates: Sex	Model fit compared to analysis with no covariates: Sex and TIV
**(A) Fear acquisition training**								
			Regression	BF_{01}	Regression	BF_{01}		
dACC	SCR	Full	$F(2,104) = 1.90, p = .15, R^2 = .04$	4.37	$F(1,105) = 1.61, p = .21, R^2 = .02$	2.38	$F(1,104) = 2.19 , p = .14$	$F(1,103) = 1.95, p = .17$
		1st half	$F(2,104) = .44, p = .65, R^2 = .01$	15.15	$F(1,105) = .62, p = .43, R^2 = .01$	3.70	$F(1,104) = .26, p = .61$	$F(1,103) = .63, p = .43$
		2nd half	$F(2,104) = 2.77, p = .07, R^2 = .03$	2.11	$F(1,105) = 1.81, p = .18, R^2 = .02$	2.18	$F(1,104) = 3.73, p = .06$	$F(1,103) = 2.35, p = .13$
	ratings	Post	$F(2,100) = 1.01, p = .37, R^2 = .02$	8.77	$F(1,101= 1.99, p = .16, R^2 = .02$	1.98	$F(1,100) = .04, p = .85$	**$F(1,99) = 5.38, p = .02$**
Amygdala	SCR	Full	$F(2,104) = 1.19, p = .31, R^2 = .02$	6.85	$F(1,105) = 2.27, p = .13, R^2 = .02$	1.78	$F(1,104) = .13, p = .72$	$F(1,103) = .42, p = .52$
		1st half	$F(2,104) = .07, p = .93, R^2 = < .01$	18.87	$F(1,105) = .12, p = .73, R^2 = < .01$	4.63	$F(1,104) = .02, p = .89$	$F(1,103) = .51, p = .48$
		2nd half	$F(2,104) = 2.36, p = .10, R^2 = .04$	2.27	**$F(1,105) = 4.55, p = .04, R^2 = .04$**	**0.65**	$F(1,104) = .21, p = .65$	$F(1,103) = .16, p = .69$
	ratings	Post	$F(2,100) = .80, p = .45, R^2 = .02$	1.03	$F(1,101) = .98, p = .33, R^2 = .01$	3.11	$F(1,100) = .64, p = .43$	$F(1,99) = 2.90, p = .09$
**(B) Extinction training**								
Amygdala	SCR	Full	$F(2,104) = .07, p = .93, R^2 < .01$	18.18	$F(1,105) = .02, p = .70, R^2 < .01$	4.59	$F(1,104) = .005, p = .95$	$F(1,103) = .003, p = .96$
		1st half	$F(2,104) = .23, p = .80, R^2 < .01$	16.39	$F(1,105) = .42, p = .52, R^2 < .01$	4.05	$F(1,104) = .03, p = .85$	$F(1,103) = .004, p = .95$
		2nd half	$F(2,104) = .09, p = .91, R^2 < .01$	19.23	$F(1,105) = .06, p = .81, R^2 < .01$	4.76	$F(1,104) = .13, p = .72$	$F(1,103) = .03, p = .86$
	ratings	pre-post	$F(2,94) = .22, p = .88, R^2 < .01$	16.13	$F(1,95) = .04, p = .84, R^2 < .01$	4.59	$F(1,94) = .40, p = .53$	$F(1,93) = .24, p = .62$
		Pre	$F(2,95) = .78, p = .46, R^2 = .02$	9.90	$F(1,96) < .01, p = .98, R^2 < .01$	4.69	$F(1,95) = 1.56, p = .22$	$F(1,94) = 1.10, p = .30$
		Post	$F(2,101) = .40, p = .67, R^2 = .01$	14.08	$F(1,102) < .01, p = .98, R^2 < .01$	4.83	$F(1,101) = .81, p = .37$	$F(1,100) = 1.06, p = .31$
mOFC	SCR	Full	$F(2,104) = .04, p = .96, R^2 < .01$	21.74	$F(1,105) = .92, p = .88, R^2 < .01$	4.83	$F(1,104)= .07, p = .81$	$F(1,103) = .04, p = .84$
		1st half	$F(2,104) = .07, p = .93, R^2 < .01$	20.83	$F(1,105) = .13, p = .72, R^2 < .01$	4.61	$F(1,104) = .02, p = .90$	$F(1,103) = .09, p = .77$
		2nd half	$F(2,104) = .05, p = .95, R^2 <.01$	21.28	$F(1,105) = .05, p = .82, R^2 < .01$	4.78	$F(1,104) = .05, p = .83$	$F(1,103) < .01, p = .95$
	ratings	pre-post	$F(2,94) = .09, p = .91, R^2 < .01$	18.87	$F(1,95) < .01, p = .98, R^2 < .01$	4.67	$F(1,94) = .18, p = .67$	$F(1,93) = .50, p = .48$
		Pre	$F(2,95) = .70, p = .50, R^2 = .01$	11.49	$F(1,96) = .11, p = .74, R^2 <. 01$	4.46	$F(1,95) = 1.28, p = .26$	$F(1,94) = 1.27, p = .26$
		Post	$F(2,101) = .78, p = .46, R^2 = .02$	11.11	$F(1,102) = .82, p = .37, R^2 = .01$	3.36	$F(1,101) = .74, p = .39$	$F(1,100) = .83, p = .37$

2.3 Robustness analyses with raw SCR

All main pre-registered hypotheses regarding the association of SCR and brain morphology were also performed with raw SCR scores instead of log-transformed and range corrected SCR scores that were included in the analysis of the main manuscript.

In brief, the analyses reveal a very similar pattern of results to that presented in the main manuscript suggesting no relationship between differential SCR during fear acquisition and extinction training and brain morphology with both traditional NHST and a Bayesian approach.

Supplementary Table 3. Results of regression analyses with cortical thickness/subcortical volume and *raw* differential SCR during fear acquisition and extinction training (controlled for sex and TIV) and Bayes factor BF_{01} providing relative evidence for intercept-only model against the regression model.

	**dACC**		**Amygdala**		**mOFC**	
**(A) Fear acquisition training**	**Regression**	**BF_{01}**	**Regression**	**BF_{01}**	**Regression**	**BF_{01}**
Differential SCR: Full phase	$F(3,103) = 2.55$, $p = .06$ $R^2 = .07$	2.31	$F(3,103) = 1.23$, $p = .3$, $R^2 = .03$	12.82	---	---
Differential SCR: First half	$F(3,103) = .94$, $p = .42$ $R^2 = .03$	18.18	$F(3,103) = .05$, $p = .98$, $R^2 < .01$	62.50	---	---
Differential SCR: Second half	$F(3,103) = 2.67$, $p = .051$, $R^2 = .07$	1.92	$F(3,103) = 1.68$, $p = .18$, $R^2 = .05$	7.04	---	---
**(B) Extinction training**						
Differential SCR: Full phase	---		$F(3,103) = .16$, $p = .92$, $R^2 < .01$	50.00	$F(3,103) = .13$, $p = .94$, $R^2 < .01$	55.56
Differential SCR: First half	---		$F(3,103) = .17$, $p = .92$, $R^2 < .01$	52.63	$F(3,103)$, .11, $p = .95$, $R^2 < .01$	55.56
Differential SCR: Second half	---		$F(3,103) = .11$, $p = .95$, $R^2 < .01$	58.82	$F(3,103) = .18$, $p = .91$, $R^2 = .01$	52.63

2.4 Robustness analyses – outliers removed

We checked the data for outliers (> 3 SD below or above mean, see for example Winkelmann et al., 2015) in fear ratings and SCR. One participant was excluded based on post-acquisition fear ratings, one based on pre-post extinction fear ratings and four based on differential SCR during extinction. The affected analyses were rerun after exclusions and the full results can be found in Supplementary Table 4. In summary, the general pattern of results remained the same with no significant associations.

Supplementary Table 4. Results of regression analyses with cortical thickness/subcortical volume and differential SCR and fear ratings during fear acquisition and extinction training (controlled for sex and TIV) with outliers (> 3 SD below or above mean) removed. Bayes factor BF_{01} provides relative evidence for intercept-only model against the regression model.

	**dACC**		**Amygdala**	
**(A) Fear acquisition training**	**Regression**	**BF_{01}**	**Regression**	**BF_{01}**
Differential post acquisition fear ratings	$F(3,98) = 1.45$, $p = .23$, $R^2 = .04$	1.08	$F(3,98) = 0.99$, $p = .40$, $R^2 = .03$	19.61
	**Amygdala**		**OFC**	
**(B) Extinction training**	**Regression**	**BF_{01}**	**Regression**	**BF_{01}**
Differential SCR: Full phase	$F(3,99) = .16$, $p = .93$, $R^2 < .01$	52.63	$F(3,99) = .25$, $p = .86$, $R^2 = .01$	45.45
Differential SCRs First half	$F(3,99) = .21$, $p = .89$, $R^2 = .01$	47.62	$F(3,99) = .78$, $p = .51$, $R^2 = .02$	23.26
Differential SCR: Second half	$F(3,99) = 1.45$, $p = .20$, $R^2 = .05$	76.92	$F(3,99) = .14$, $p = .94$, $R^2 < .01$	52.63
Differential fear ratings [pre-post extinction]	$F(3,92) = .28$, $p = .84$, $R^2 = .01$	41.67	$F(3,92) = .33$, $p = .81$, $R^2 = .01$	38.46

3. Additional, non-pre-registered analyses aiming to (conceptually) replicate previously reported findings

3.1 No association of dACC cortical thickness and SCR to the CS+ and CS- during acquisition training

In a non-pre-registered analysis we aimed to replicate the previous finding of a significant correlation between dACC thickness and SCR to the CS+ but not the CS- during fear acquisition training (Milad et al., 2007). To be consistent with our previous analyses, we additionally computed partial correlations with sex as well as sex and TIV.

None of these analyses revealed a significant correlation between dACC thickness and SCR to either the CS+ or the CS- irrespective of covariates included and Bayes factors provide further evidence for the null hypothesis (for full results see Supplementary Table 5 and Supplementary Figure 4).

Supplementary Table 5. (Partial) Correlations between dACC thickness and CS+ and CS- and Bayes factor BF_{01} providing relative evidence for the full correlation against a null model.

	dACC		dACC[a]		dACC[b]		
	r	*p*	*r*	*p*	*r*	*p*	BF_{01}
CS+	.11	.27	.16	.11	.16	.10	2.51
CS-	.04	.65	.08	.39	.08	.43	4.07

Note. [a] corrected for sex, [b] corrected for sex and TIV

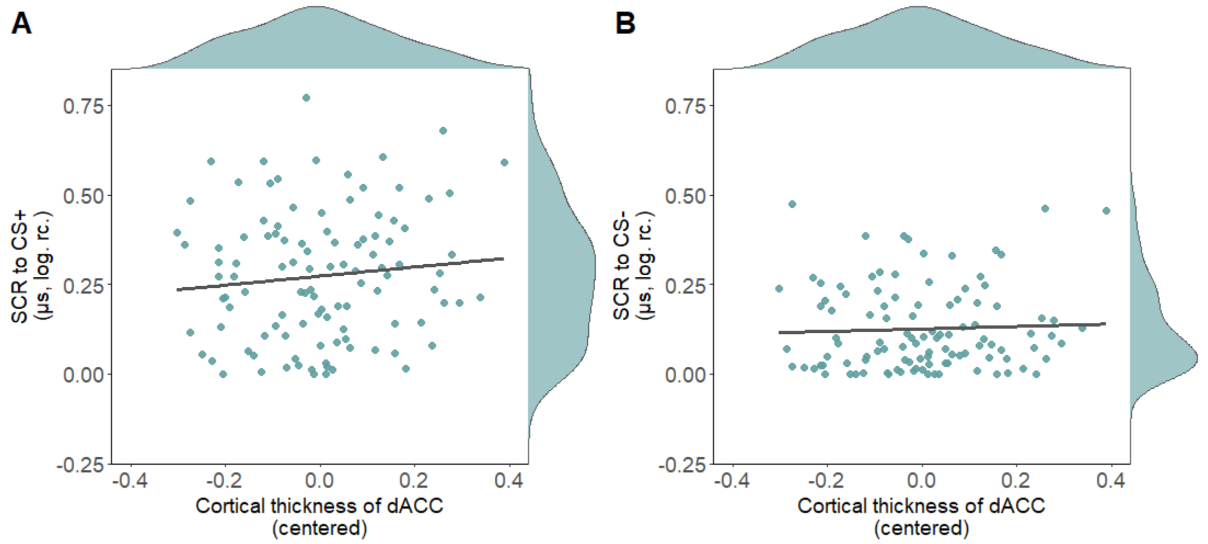

Supplementary Figure 4. Scatterplots with marginal densities illustrating the (absence of) associations between dACC thickness and SCR during acquisition training (A) to the averaged CS+ trials and (B) the averaged CS- trials.

3.2 No association between thickness of the insula and differential SCR and ratings during fear acquisition and extinction

Hartley et al. (2011) reported a positive correlation between right (posterior) insula thickness and differential SCRs during acquisition training – even though in one out of two data sets, the correlation did not survive correction for multiple comparisons. In the current study, we aimed to replicate this finding in a substantially larger sample and for completeness extend them to differential fear ratings as well as extinction training. These analyses were not pre-registered.

We did not observe any significant correlations between differential SCRs or post acquisition ratings during acquisition training (see Supplementary Figure 5A and B) or differential SCRs and ratings (pre – post extinction) during extinction (Supplementary Figure 5C and D) for either right or left hemisphere or averaged insula thickness (for full results see Supplementary Table 6). Bayes factors further provide moderate evidence for the null hypothesis.

Supplementary Table 6.
Correlations between thickness of the insula (averaged over both hemispheres, left and right) with differential SCRs and differential ratings (post acquisition and pre – post extinction) during acquisition and extinction training and Bayes factor BF_{01} providing relative evidence for the null model against the tested correlation.

	**Left Insula**			**Right Insula**			**Averaged Insula**		
**(A) Fear acquisition training**									
	r	***p***	**BF_{01}**	***r***	***p***	**BF_{01}**	***r***	***p***	**BF_{01}**
Differential SCR	.03	.79	4.35	.12	.21	2.14	.08	.43	3.34
Differential post acquisition fear ratings	.02	.84	4.33	.07	.50	3.55	.05	.64	3.98
**(B) Extinction training**									
Differential SCR	.03	.74	4.27	.04	.72	4.22	.04	.70	4.18
Differential fear ratings [pre-post extinction]	.06	.26	3.65	.05	.63	3.82	.06	.54	3.60
Differential pre extinction fear ratings	.03	.74	4.08	.12	.25	2.29	.08	.43	3.23
Differential post extinction fear ratings	-.04	.72	4.17	.13	.18	1.89	.04	.67	4.07

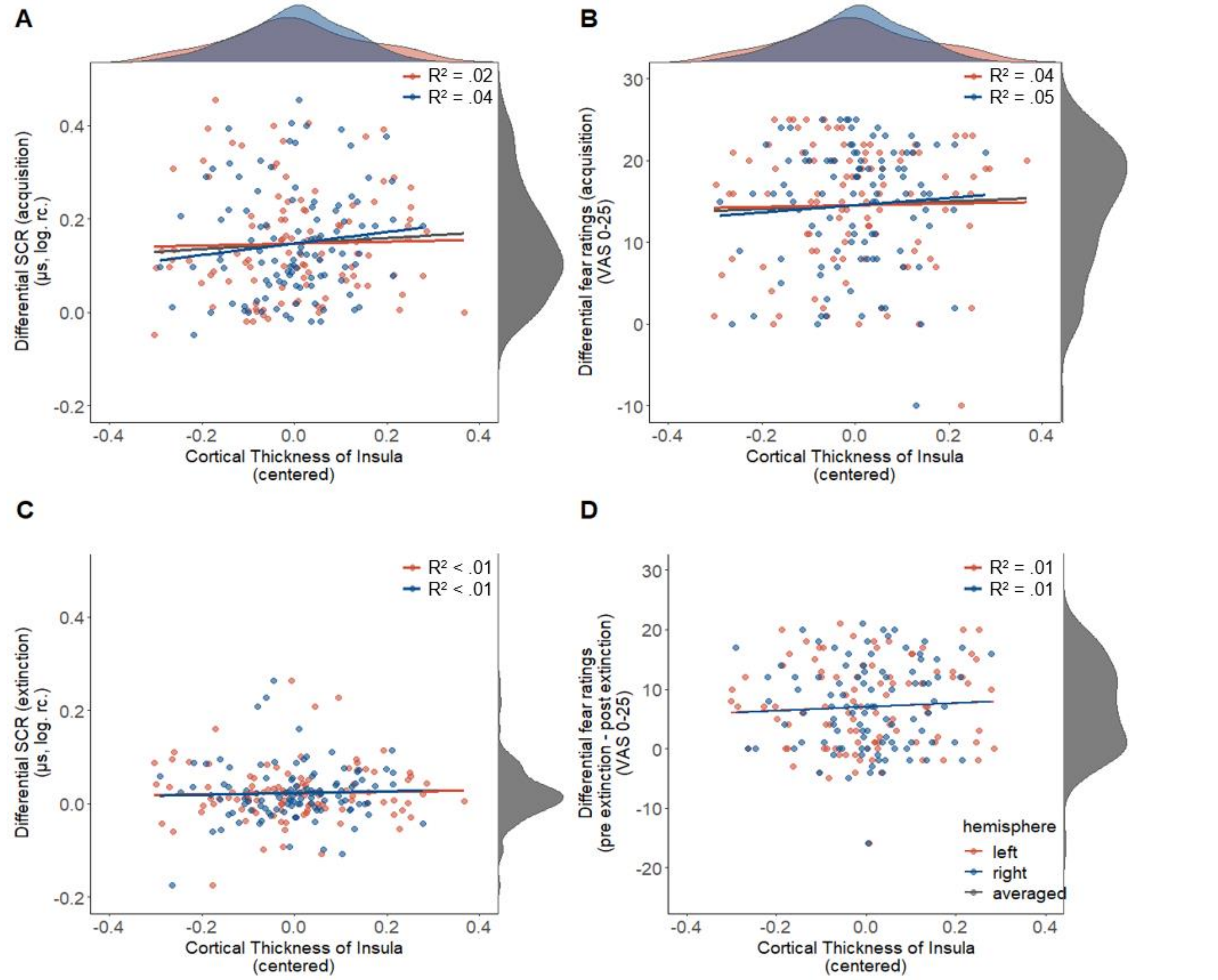

Supplementary Figure 5. Scatterplot with marginal densities illustrating the (absence of) associations between (A) differential SCR during and (B) differential fear ratings after acquisition training as well as (C) differential SCR during and (D) differential pre - post extinction fear ratings [[($CS+_{pre}$)-($CS-_{pre}$)] - [($CS+_{post}$)-($CS-_{post}$)]] and left (red), right (blue) insula as well as averaged between hemispheres (dark grey regression line only).

3.3 No association of amygdala volume with trait and state anxiety

Previously, a *negative* correlation between left amygdala volume and state and trait anxiety (Blackmon et al., 2011), as well as a *positive* correlation between left amygdala volume and trait anxiety (Baur et al., 2012) has been reported while a third study (Winkelmann et al., 2015) did not observe any association between amygdala volume and trait anxiety.

In the current study, we adopted the approach of Winkelmann et al. (2015) and calculated partial correlations between amygdala volume and trait anxiety as well as state anxiety (assessed prior to Day 1 acquisition training and Day 2 extinction training respectively) while controlling for the pre-registered covariates age, sex and TIV. We did not observe any significant associations between state or trait anxiety and averaged amygdala volume or right or left amygdala volume (for full results see Supplementary Table 7 and Supplementary Figure 6), which is further supported by Bayes factors suggesting support for the null hypothesis.

Supplementary Table 7.
Partial correlations of subcortical volume and STAI Trait/STAI State as indicator for anxiety and Bayes factor BF_{01} providing relative evidence for the null model against the full correlation.

		**Left Amygdala**[a]			**Right Amygdala**[a]			**Averaged Amygdala**[a]		
	M (SD) [range]	***r***	***p***	**BF_{01}**	***r***	***p***	**BF_{01}**	***r***	***p***	**BF_{01}**
STAI Trait	34.61 (7.19) [24, 55]	.09	.40	4.10	.02	.83	2.92	.06	.55	3.53
STAI State (Day 1)	35.25 (5.25) [24, 48]	.05	.64	3.36	.10	.35	3.65	.07	.45	3.44
STAI State (Day 2)	35.53 (6.77) [23, 52]	.04	.71	3.79	.06	.58	3.79	.05	.61	3.75

Note. [a] corrected for age, sex and TIV

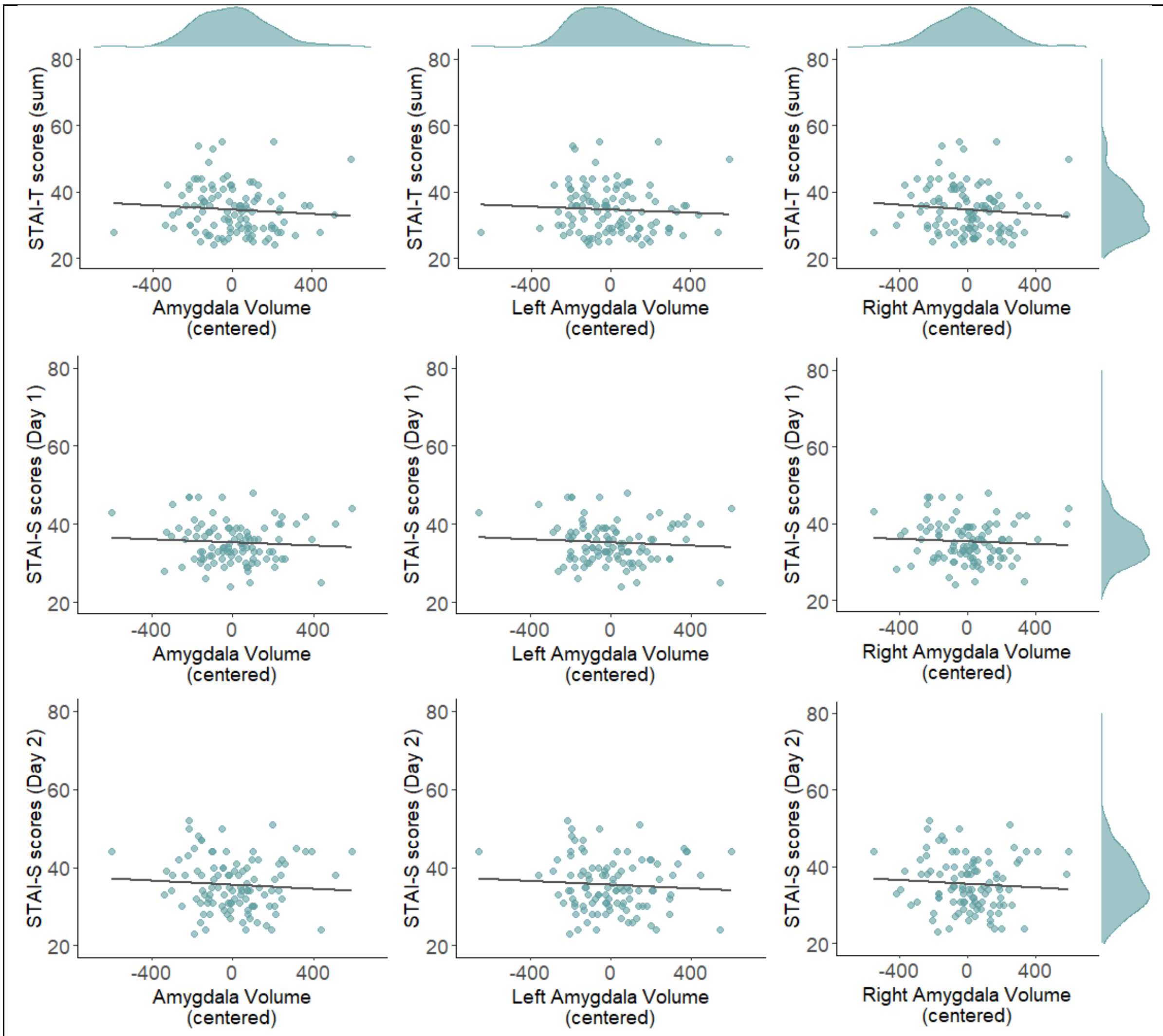

Supplementary Figure 6. Scatterplots with marginal densities illustrating the lack of an association between trait anxiety (STAI-T) as well as state anxiety (STAI-S) prior to acquisition training (Day 1), and prior to extinction training (Day2) and amygdala volume (centered, for averaged, left and right volume).
